# Overexpression of a Tartary Buckwheat Gene, *FtbHLH3*, Enhances Drought/Oxidative Stress Tolerance in Transgenic *Arabidopsis*

**DOI:** 10.3389/fpls.2017.00625

**Published:** 2017-04-25

**Authors:** Pan-Feng Yao, Cheng-Lei Li, Xue-Rong Zhao, Mao-Fei Li, Hai-Xia Zhao, Jin-Ya Guo, Yi Cai, Hui Chen, Qi Wu

**Affiliations:** College of Life Science, Sichuan Agricultural UniversityYa'an, China

**Keywords:** tartary buckwheat, bHLH protein, drought stress, antioxidant system, chlorophyll fluorescence, transgenic *A. thaliana*

## Abstract

bHLH (basic helix-loop-helix) transcription factors play important roles in the abiotic stress response in plants, but their characteristics and functions in tartary buckwheat (*Fagopyrum tataricum*), a flavonoid-rich cereal crop with a strong stress tolerance, have not been fully investigated. Here, a novel bHLH gene, designated *FtbHLH3*, was isolated and characterized. Expression analysis in tartary buckwheat revealed that *FtbHLH3* was mainly induced by polyethylene glycol 6000 (PEG6000) and abscisic acid (ABA) treatments. Subcellular localization and a yeast one-hybrid assay indicated that FtbHLH3 has transcriptional activation activities. Overexpression of *FtbHLH3* in *Arabidopsis* resulted in increased drought/oxidative tolerance, which was attributed to not only lower malondialdehyde (MDA), ion leakage (IL), and reactive oxygen species (ROS) but also higher proline (Pro) content, activities of antioxidant enzymes, and photosynthetic efficiency in transgenic lines compared to wild type (WT). Moreover, qRT-PCR analysis indicated that the expression of multiple stress-responsive genes in the transgenic lines was significantly higher than in WT under drought stress. In particular, the expression of *AtNCED*, a rate-limiting enzyme gene in ABA biosynthesis, was increased significantly under both normal and stress conditions. Additionally, an ABA-response-element (ABRE) was also found in the promoter regions. Furthermore, the transgenic *Arabidopsis* lines of the *FtbHLH3* promoter had higher GUS activity after drought stress. In summary, our results indicated that *FtbHLH3* may function as a positive regulator of drought/oxidative stress tolerance in transgenic *Arabidopsis* through an ABA-dependent pathway.

## Introduction

Drought is one of the harshest environmental factors during the plant growth process, limiting plant development and crop productivity and further leading to large economic losses (Umezawa et al., 2006). To resist the environmental stress factors, plants have evolved a variety of complex mechanisms. The interaction of multiple mechanisms increases the biosynthesis of functional and structural protectants, including osmolytes and antioxidants, or stress-tolerance-conferring proteins. Various functional proteins involved in these processes are mainly regulated by various types of transcription factors (TFs), such as the MYB, bHLH, DREB, and WRKY families. These transcription factors play crucial roles in the signaling network of plant resistance to environmental stimulation (Wang et al., 2011; Huang et al., 2012).

bHLH is one of the largest family of transcription factors in plants and has been divided into 26 subgroups (Pires and Dolan, 2010). Maize regulatory gene (*R*) was the first cloned transcription factor of the bHLH family (Ludwig et al., 1989), and since then, many bHLH factors involved in the responses to abiotic stresses have been characterized in different plants. In *Arabidopsis, AtbHLH44* and *AtbHLH122* function in response to drought stress by reducing the expression of genes encoding PP2Cs (Jung et al., 2008; Liu et al., 2014). In *Vitis vinifera*, overexpression of *VvbHLH1* increased the tolerance of transgenic *Arabidopsis* to salt and drought tolerance via increasing the total content of flavonoids (Wang et al., 2016b). In *Oryza rufipogon*, several bHLH TFs were also involved in response to abiotic stresses. For example, the rice transcription factor *OrbHLH2* regulated salt-stress signals independent of ABA in *Arabidopsis* by upregulating the expression of stress-responsive genes DREB1A/CBF3, RD29A, COR15A, and KIN1 (Zhou et al., 2009). Ectopic expression of *OrbHLH001* improved the tolerance of transgenic plants to freezing and salt stresses, and the function is independent of the CBF/DREB1 cold-response pathway (Li et al., 2010). Thus, bHLH transcription factors play significant roles in the molecular mechanism of stress resistance and improve the drought resistance of crops.

Tartary buckwheat (*Fagopyrum tataricum*) is a dicotyledonous crop belonging to the Polygonaceae family. Buckwheat, a pseudocereal, is recognized as a health food as it contains numerous flavonoids with antioxidant activity (Thwe et al., 2014). Meanwhile, because of its strong abiotic resistant nature, tartary buckwheat is cultivated as an important crop in the high mountain areas of western China and in the Himalayan hills (up to 4,500 m high-altitude areas). Recent studies on the stress resistance of TFs in tartary buckwheat have mainly focused on MYBs. Ectopic expression of *FtMYB12* enhanced the tolerance of transgenic *A. thaliana* to cold stress (Zhou et al., 2015). Meanwhile, eight R2R3-MYB TFs, which may regulate the abiotic stress response in plants (Gao et al., 2016), were identified and preliminarily validated. However, research on bHLH factors from tartary buckwheat involved in abiotic stress is limited, and more studies cloning and characterizing their functions are necessary. In this study, a drought-responsive bHLH gene, *FtbHLH3*, was isolated and characterized from tartary buckwheat. Our results indicated that *FtbHLH3* improved the tolerance of transgenic plants to drought/osmotic stress through activating the antioxidant system and upregulating the expression of multiple metabolic pathway genes.

## Materials and methods

### Plant materials and stress conditions

Tartary buckwheat, called “Xiqiao No. 2,” was cultivated in a farm at Sichuan Agriculture University, and samples were collected to clone and detect the expression profiles of *FtbHLH3* after multiple abiotic stresses. *Arabidopsis thaliana* (ecotype Columbia-0) was grown in an artificial climate chamber at 25°C and approximately 60% humidity (16 h light/8 h dark). Seven-day-old seedlings of tartary buckwheat were treated with the following conditions: 100 μM abscisic acid (ABA), 1 mM salicylic acid (SA), 302 nm UV-B light (302 nm, 0.1 mW/cm2), 4°C, 150 mM NaCl, and 30% PEG6000 (Su et al., 2014; Zhou et al., 2015), separately. For all of the treatments, the seedlings were collected at 0, 6, 12, 24, and 48 h (0 h was a non-treated control), and frozen in liquid nitrogen for further study. Three independent biological replicates were measured for each sample.

### Isolation and characterization of *ftbHLH3*

Total RNA was extracted from the samples using an RNAout kit (Tiandz, Beijing, China), and cDNA was synthesized with a RevertAid First Strand cDNA Synthesis kit (MBI, USA). Based on the flowering tartary buckwheat transcriptome database constructed by our laboratory (data not shown), a novel bHLH gene (designated as *FtbHLH3*) was isolated using specific primers. The gene sequence was analyzed via the NCBI database. Multiple amino acid sequence alignments were performed using ClustalX, and a phylogenetic tree was constructed using the neighbor-joining (NJ) method. The primers are listed in Supplementary Table S1.

### Subcellular localization of the ftbHLH3 protein

To determine its exact subcellular location, the coding region of *FtbHLH3* was amplified using primers incorporating the *BamH*I and *Sma*I sites at their terminus and ligated into the HBT95-GFP vectors with the same polyclonal site to produce FtbHLH3-GFP fusion proteins. Recombinant plasmids FtbHLH3-GFP and HBT95-GFP (used as a negative control) were separately transferred into the *Arabidopsis* protoplasts as previously described (Schirawski et al., 2000). The transformed cells were incubated at 22°C for 24 h in darkness and photographed using a laser confocal scanning microscope (Leica DM IRBE).

### Transcriptional assay

For the transactivation assay, the coding region of *FtbHLH3* was PCR-amplified using the following primers 5′-CGCGGATCCATGGAGGTAAATGAAGATGGGTT-3′ (*BamH*I site underlined) and 5′-AACTGCAGCATATACTTTCCTCCATAACCTGCATT-3′ (*Pst*I site underlined) and cloned into the pBridge vector to create the pBridge-FtbHLH3 constructs. The pBridge-FtbHLH3, the negative control pBridge (pBridge is the empty vector), and the positive control pBridge-GmMYBJ6 (GmMYBJ6 has transcriptional activation activities) plasmids were transformed into the AH109 yeast strain. The transformed cells were screened in SD/-His-Trp medium. Then, their *LacZ* activities were identified using the galactosidase filter lift assay.

### Generation of transgenic *Arabidopsis*

The coding region of the *FtbHLH3* gene was PCR-amplified from tartary buckwheat using the following primers 5′-GGGGTACCATGGAGGTAAATGAAGATGGGTTT-3′ (*Kpn*I site underlined) and 5′-CGGGATCCCATATACTTTCCTCCATAACCTGC-3′ (*BamH*I site underlined), and the sequence was attached to the vector pCAMBIA1301. The recombinant vector pCAMBIA1301-*FtbHLH3* was transformed into *Arabidopsis* Col-0 through the *Agrobacterium tumefaciens* strain GV3101 (Clough and Bent, 1998). The first generation (T0) seeds of transgenic plants were collected and screened in 1/2 MS medium with 50 mg L^−1^ hygromycin. Hygromycin-resistant plants were transferred into soil pots and grown in a growth chamber with a 16 h photoperiod cycle at 25°C. Finally, transgenic *Arabidopsis* plantlets were identified by PCR amplification. The T3 homozygous positive lines were used for all further experiments.

### Stress tolerance of transgenic *Arabidopsis*

For the osmotic stress tolerance assay, transgenic *A. thaliana* T3 and WT were used *in vitro*. Sterilized seeds were germinated in 1/2 MS medium under a 16 h photoperiod cycle at 25°C, and 4-day-old seedlings were transplanted to 1/2 MS medium supplemented with 150 and 250 mM mannitol. The germination rate, root length, content of hydrogen peroxide (H_2_O_2_), and SOD, CAT and POD activities were measured as previously described (Maehly and Chance, 1954; Beyer and Fridovich, 1987). For the drought stress tolerance assay, positive lines and WT plants were sown in pots and regularly watered for 3 weeks. At 4 weeks, plants with a similar growth status were subjected to water withholding for 30 days. The survival rate was measured after 30 days. Meanwhile, proline (Pro), malondialdehyde (MDA), ion leakage (IL), hydrogen peroxide (H_2_O_2_), superoxide anion radicals (${\text{O}}_{2}^{-}$), peroxidase (POD), catalase (CAT), and superoxide dismutase (SOD) activities were detected for 15 or 30 days in the transgenic *A. thaliana* and WT plants (Heath and Packer, 1968; Pan et al., 2012). For the oxidative stress assay, the leaves of transgenic and WT plants were incubated on liquid MS medium and MS medium containing different concentrations of methyl viologen (MV) (10, 20, and 30 μM) for 32 h in the light. The chlorophyll and H_2_O_2_ content and antisuperoxide anion activity (an index indicating superoxide anion levels) were determined after treatment. Simultaneously, 5-week-old transgenic and WT lines were subjected to 20 μM MV for 15 days. Afterward, leaves were collected to detect chlorophyll fluorescence, DAB and NBT staining, and SOD, CAT, and POD activities (Beyer and Fridovich, 1987; Maehly and Chance, 1954). Furthermore, the expression levels of *AtSOD, AtCAT* and *AtPOD* were detected under the same treatment condition. Each sample contained three replicates.

### Cloning of the *FtbHLH3* promoter and GUS histochemistry of transgenic *Arabidopsis* under drought stress

The 5′-upstream promoter region of *FtbHLH3* was isolated according to the Genome Walking Kit (TaKaRa, Japan) with nested specific primers sp1, sp2, sp3, and AP primers provided in the kit. The PCR product was subcloned into a pMD™19-T (TaKaRa, Japan) and sequenced. The possible putative regulatory elements of the *FtbHLH3* promoter region were identified using the PLACE/Signal Scan database.

The obtained promoter region, designated FtbHLH3P, was amplified from *F. tataricum* genomic DNA using the specific primers 3P_f_ and 3P_r_ and cloned into the plant expression vector pBI101-GUS. The recombinant plasmids FtbHLH3P-GUS and positive cauliflower mosaic virus (CaMV) 35S-GUS were introduced into *Arabidopsis* using the *Agrobacterium tumefaciens* strain GV3101, and positive lines were selected using kanamycin (50 mg L^−1^). The positive lines with FtbHLH3P:GUS and WT plants were grown in pots for 3 weeks, and 4-week-old seedlings were subjected to water withholding for a week. Histochemical analysis of GUS activity was performed.

### Quantitative real-time PCR (qRT-PCR) analysis

Total RNA was extracted using the RNeasy Plant Mini Kit (Qiagen, Valencia, CA, USA), and the purity was determined by spectrophotometry. The first-strand cDNA was synthesized using the Revert Aid™ First Strand cDNA Synthesis Kit (MBI, US). Each reaction contained 5 μL of SYBR Green II Mix, 1 μL of cDNA sample, 1 μL of primer, and 3 μL of double distilled water. The PCR protocol was as follows: 95 °C for 30 s and then 39 cycles of 95 ° C for 5 s, and 60 °C for 30 s. The housekeeping genes *FtH3* (ID: HM628903) and β*-actin* (ID: NM_112764) were used as internal standard genes in tartary buckwheat and *Arabidopsis thaliana*, respectively. The data were evaluated using the 2 ^−ΔΔCT^ method (Livak and Schmittgen, 2001). Three independent biological replicates were measured for each sample.

### Statistical analysis

Statistical analyses were performed with SPSS software (SPSS 13.0). Analysis of variance was used to compare the significant difference based on Student's *t*-test (*n* = 4).

## Results

### Cloning and characterizations analysis of *FtbHLH3*

**Figure 1:**
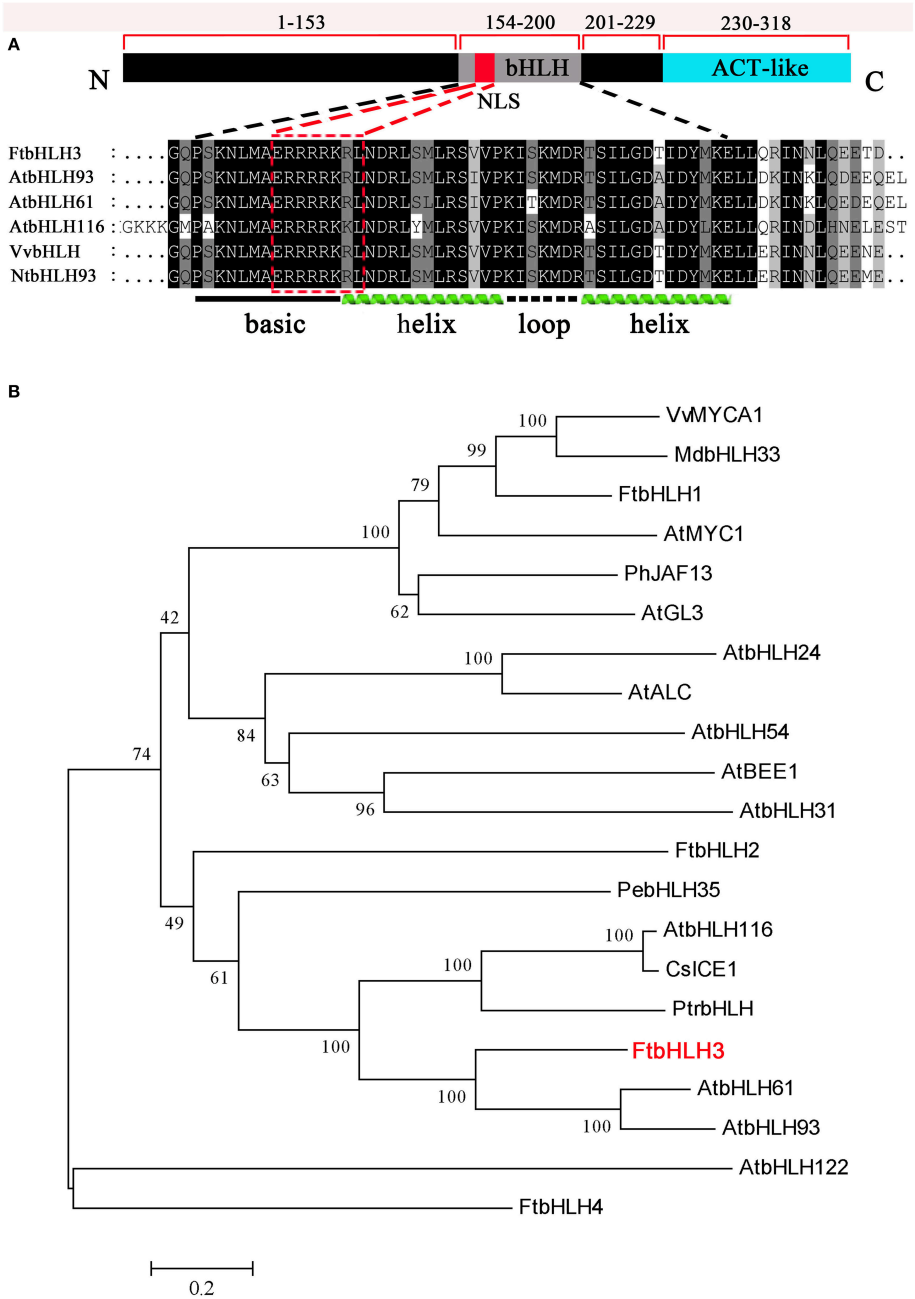
**Analysis of the structure and function of the FtbHLH3 protein. (A)** Comparison of FtbHLH3 with other known bHLH proteins using the ClustalX software. An ACT-like region, a putative NLS, and a bHLH conserved domain are indicated. The accession numbers of these proteins in GenBank are FtbHLH3 (KU296217) from *Fagopyrum tataricum*, AtbHLH93 (AED98080) from *Arabidopsis thaliana*, AtbHLH61 (AF488594) from *Arabidopsis thaliana*, AtbHLH116 (Y079016) from *Arabidopsis thaliana*, VvbHLH (AFR78197) from *Vitis vinifera*, NtbHLH93 (XP_016487716) from *Nicotiana tabacum*. **(B)** Phylogenetic relationship of FtbHLH3 with other bHLH proteins. The GenBank accession numbers are VvMYCA1 (EF193002) from *Vitis vinifera*, MdbHLH33 (ABB84474) from *Malus domestica*, FtbHLH1 (KT737454) from *Fagopyrum tataricum*, AtMYC1 (NP_191957) from *Arabidopsis thaliana*, PhJAF13 (AAC39455) from *Petunia hybrida*, AtGL3 (Q9FN69) from *Arabidopsis thaliana*, AtbHLH24 (Q9FUA4) from *Arabidopsis thaliana*, AtALC (OAO95101) from *Arabidopsis thaliana*, AtbHLH54 (Q8LEG1) from *Arabidopsis thaliana*, AtBEE1 (OAP12570) from *Arabidopsis thaliana*, AtbHLH31 (Q0JXE7) from *Arabidopsis thaliana*, FtbHLH2 (KT737455) from *Fagopyrum tataricum*, PebHLH35 (KJ363186) from *Populus euphratica*, AtbHLH116 (Y079016) from *Arabidopsis thaliana*, CsICE1 (XP_010514336) from *Camellia sativa*, PtrbHLH (AFY17139) from *Poncirus trifoliata*, FtbHLH3 (KU296217) from *Fagopyrum tataricum*, AtbHLH61 (AF488594) from *Arabidopsis thaliana*, AtbHLH93 (AED98080) from *Arabidopsis thaliana*, AtbHLH122 (Q9C690) from *Arabidopsis thaliana*, FtbHLH4 (ALY11153) from *Fagopyrum tataricum*.

To identify putative bHLH genes that may be closely involved in the abiotic stresses, two bHLH genes *AtbHLH116* and *AtbHLH61* as probes were screened from the tartary buckwheat transcriptome database constructed by our laboratory. According to a set of unigenes with annotation as bHLH TFs, more than 4 full length bHLH genes were cloned. Of them, the gene (*FtbHLH3*) encoded a bHLH factor which was clustered with the PebHLH35 factor involved in drought stress in *Populus euphratica* (Dong et al., 2014). The *FtbHLH3* gene encodes a protein of 318 amino acid residues with a predicted molecular mass of 36.12 kDa and a calculated pI of 4.89. *FtbHLH3* has an 957 bp open reading frame (GenBank Accession No. KU296217). The predicted FtbHLH3 protein contained a putative NLS sequence. The multiple sequence alignment results implied that a sequence rich in acidic amino acids is present at its C-terminal region (Figure 1A). Phylogenetic analysis showed that FtbHLH3 was classified with stress-related bHLH TFs from other plants into the same cluster belonging to the subgroup III of *Arabidopsis* bHLH (Zhao et al., 2013). That means FtbHLH3 might have the similar functions involved in stress responses in plant (Figure 1B).

**Figure 2:**
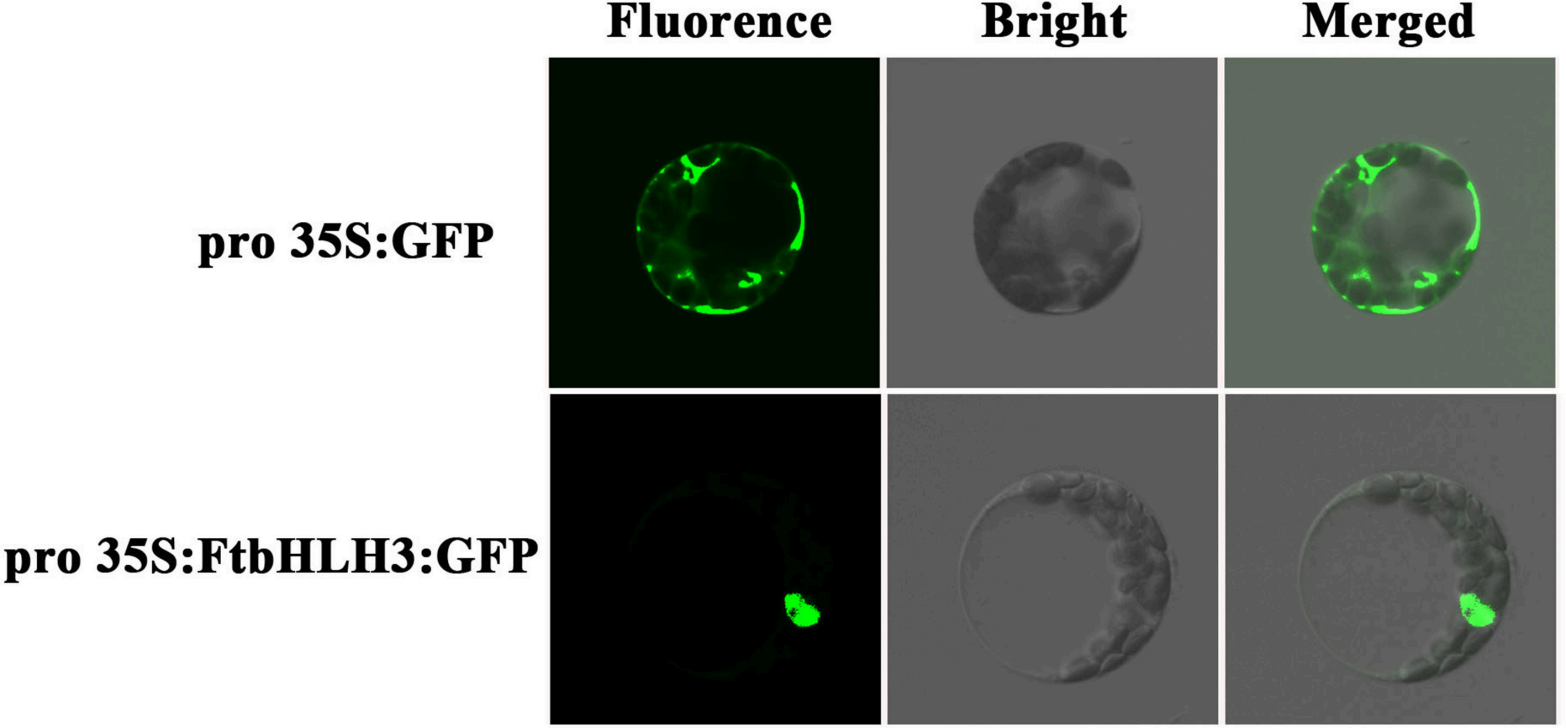
**Subcellular localization of the 35S:FtbHLH3:GFP fusion protein in *Arabidopsis* protoplasts**. *Arabidopsis* protoplasts transformed with 35S:GFP were used as a control.

**Figure 3:**
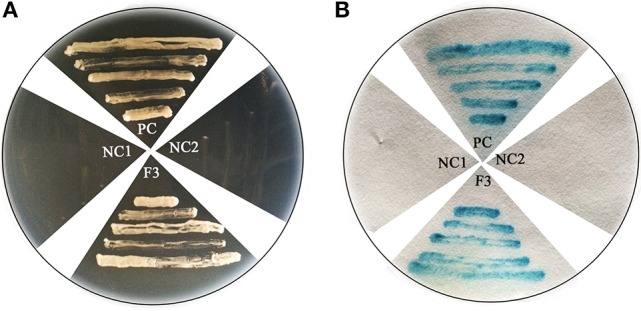
**Transactivation activity verification of FtbHLH3 in yeast. (A)** The transformed cells were screened onto SD/-His-Trp medium. **(B)** After the galactosidase filter lift assay. F3: pBridge-bHLH3; Positive Control (PC): pBridge-G*m*MYBJ6; Negative Control 1(NC1): empty pBridge plasmid; Negative Control 2 (NC2): AH109 cells. Fusion proteins of pBridge-FtbHLH3, pBridge-GmMYBJ6 and pBridge were expressed in the yeast strain AH109.

To further verify the characterization of FtHLH3 as a transcription factor, 35S:FtbHLH3:GFP was transiently expressed in *Arabidopsis* protoplast cells. Figure 2 showed that the 35S:GFP control displayed fluorescence throughout the whole cell. By contrast, the strong green fluorescence was very localized in FtbHLH3:GFP transformed cell, suggesting that FtbHLH3 could be located in the nuclear. Moreover, the transcriptional activity of FtbHLH3 was analyzed using a yeast assay system. Yeast cells containing pBridge-FtbHLH3 and pBridge-GmMYBJ6 (positive control) grew well in SD/-Trp/-His medium, whereas cells containing empty pBridge (negative control) did not grow (Figure 3A). The result indicates that FtbHLH3 activated the transcription of reporter gene *His* in the yeast AH109 strain. Meanwhile, there was no color change in the negative control, but the positive control and pBridge-FtWD40 had a significant blue reaction after the treatment with X-gal (Figure 3B). The above results indicate that FtbHLH3 is a transcriptional activator.

### *FtbHLH3* transcript is induced by PEG6000 and ABA treatments

To determine the expression patterns of *FtbHLH3* in different tissue, qRT-PCR was carried out with mRNA from different tissues. *FtbHLH3* was expressed in all tissues examined including root, stem, leaf and flower. Its transcription level in the flower was 6.09-, 1.41-, and 1.89-fold compared to that in the root, stem and leaf, respectively (Supplemental Figure S1). To investigate the responses of *FtbHLH3* to multiple abiotic stresses, 4°C, ABA, NaCl, PEG6000, SA, and UV-B treatments were applied. As a whole, the *FtbHLH3* gene showed different expression patterns under various abiotic stress treatments. Under PEG treatment, the expression of the *FtbHLH3* gene rapidly increased and reached a maximum value (14.56-fold) at 12 h, then tended to be high with little alteration (Supplemental Figure S2). Meanwhile, expression of *FtbHLH3* was also induced by 4°C and ABA treatment, but the highest levels of transcription occurred at 24 h (Supplemental Figure S2). Under UV-B treatment, the *FtbHLH3* expression increased slowly at the early stages (within 12 h), reached peak at 24 h, and then remained relatively stable (Supplemental Figure S2). In addition, The NaCl and SA treatments had no significant effect on the expression of *FtbHLH3* gene (Supplemental Figure S2). In general, PEG had more pronounced effects than the other treatments.

### Generation of transgenic *Arabidopsis*

To further characterize the function of *FtbHLH3* under drought conditions, transgenic *Arabidopsis* plants with *oxFtbHLH3* were generated. A total of 10 transgenic lines (T1) were obtained with hygromycin-resistance (50 mg L^−1^) and PCR identification (Supplemental Figure S3A). The results indicated that *FtbHLH3* was expressed in all of the transgenic plants but not in WT, and we selected three higher *FtbHLH3* expression lines (#3, #5, and #9) for subsequent analysis (Supplemental Figure S3B). The selected transgenic lines produced T3 homozygous generation, which was used for the following experiments.

### Overexpression of *FtbHLH3* enhances the tolerance of transgenic *Arabidopsis* to osmotic stress

**Figure 4:**
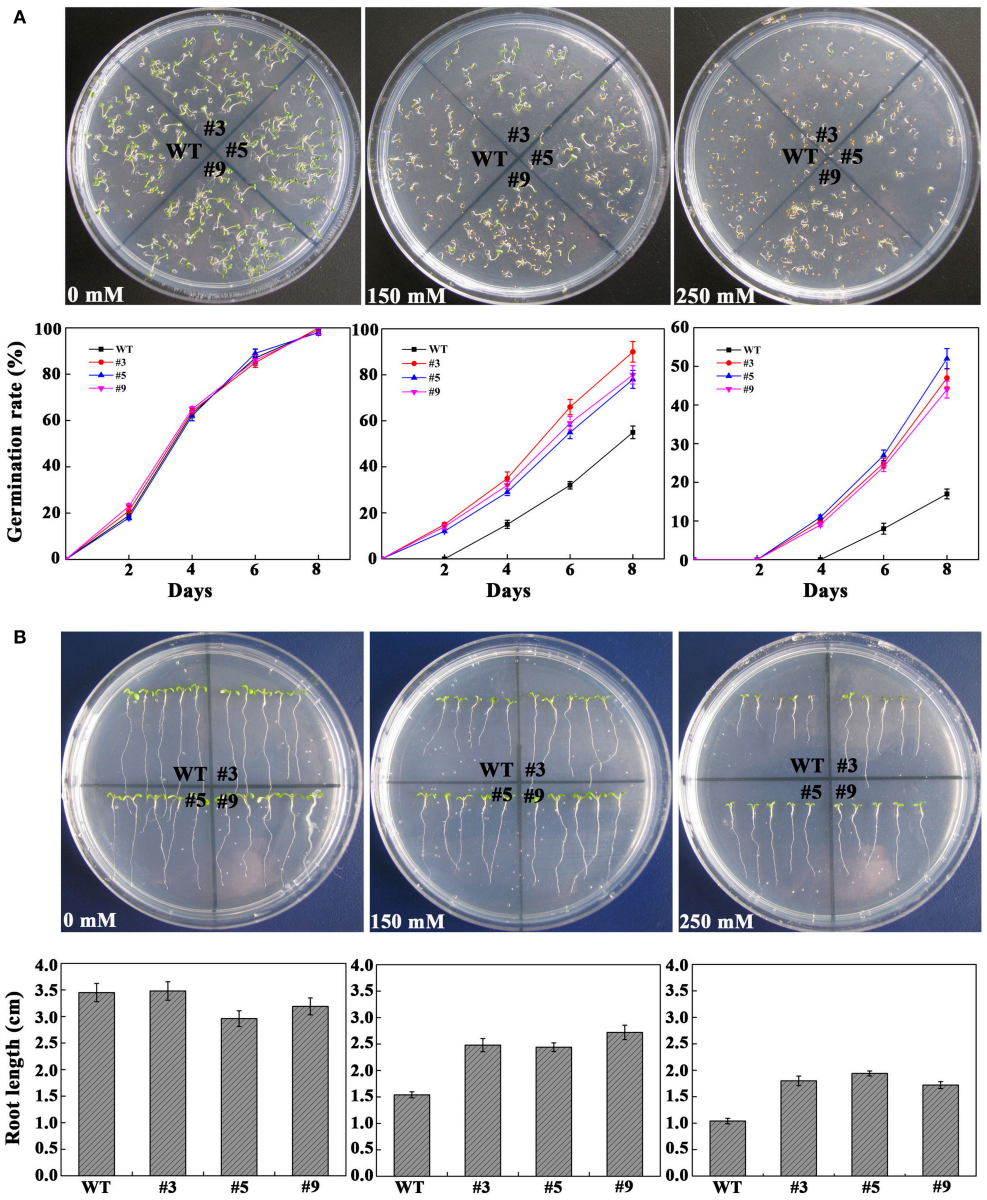
**Osmotic stress analysis of transgenic plants with *FtbHLH3*. (A)** Photographs of transgenic lines and WT seeds germinated on MS medium or 1/2 MS medium with 150 or 250 mM mannitol for 8 days. **(B)** 4-day-old seedlings were transplanted to 1/2 MS medium containing with 150 and 250 mM mannitol. Each value represents the average of the three replicates, and *error bars* represent ±SD.

**Figure 5:**
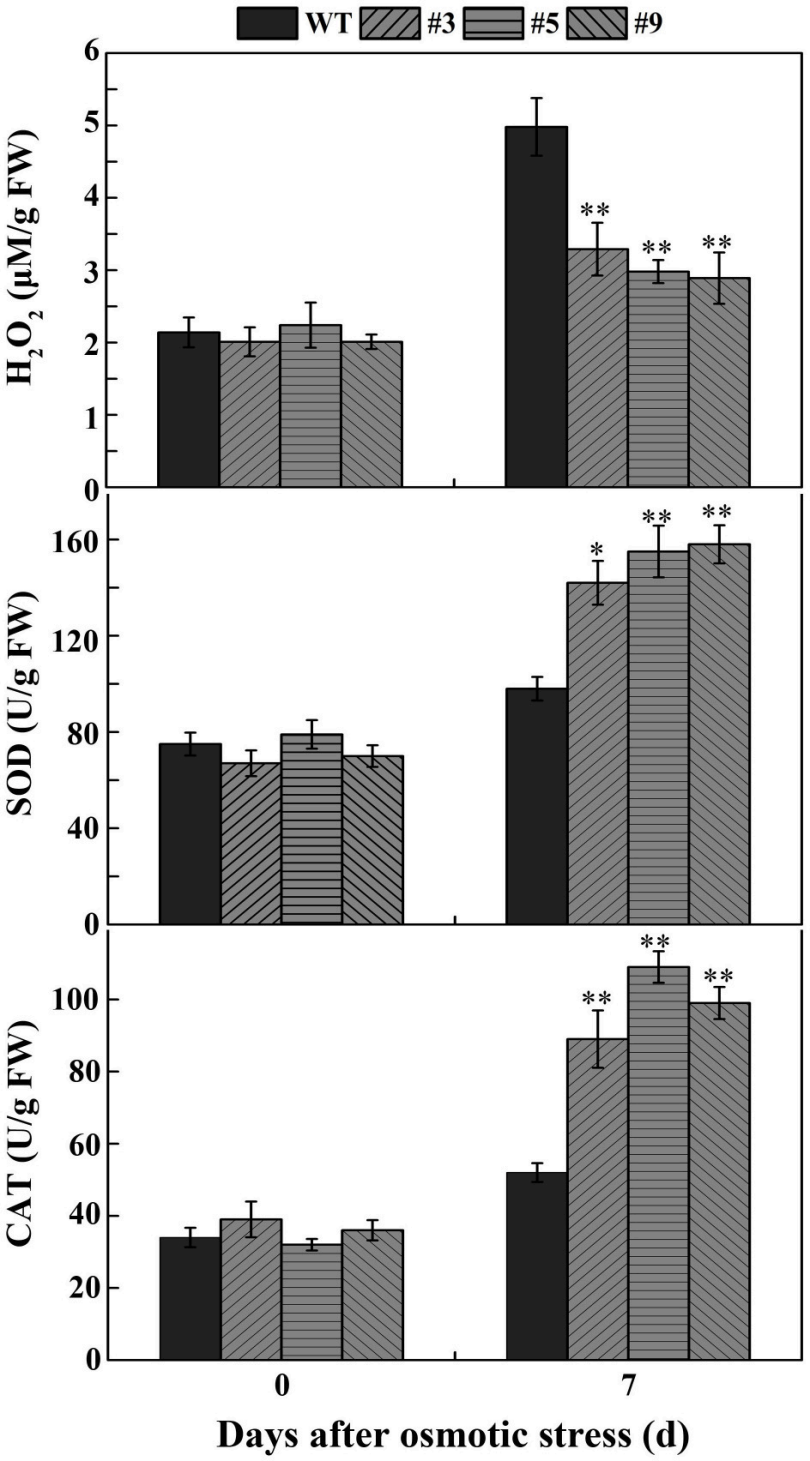
**The H_2_O_2_ accumulation and the activity of SOD and CAT in transgenic lines and WT under normal conditions and osmotic conditions**. Each value is the average of three replicates, and *error bars* represent ±SD. ^*^ and ^**^ represent significant differences between transgenic lines and WT at *P* < 0.05 and *P* < 0.01, respectively.

To confirm whether *FtbHLH3* was involved in osmotic stress, the germination rate was measured on mannitol. When the seeds were sown on the 1/2 MS medium containing mannitol, the positive plants' germination rates were clearly higher than that of the WT plants, and the difference was more significant with an increase in the mannitol concentration (Figure 4A). However, there was almost no difference under normal growth conditions. In the follow-up experiment, the length of the root was measured under the same previous treatment condition. After mannitol treatment, transgenic lines showed a significantly longer root compared to WT plants (Figure 4B). In addition, the difference was more obvious with an increased concentration. Furthermore, to understand the role of *FtbHLH3* in scavenging ROS, the H_2_O_2_ content and activities of antioxidant enzymes in transgenic lines and WT plants were detected after 7 days of mannitol treatment. The results showed that transgenic lines contained lower H_2_O_2_ accumulation and higher SOD and CAT activities than WT plants (Figure 5).

### Overexpression of *FtbHLH3* improves the tolerance of transgenic *Arabidopsis* to drought stress

**Figure 6:**
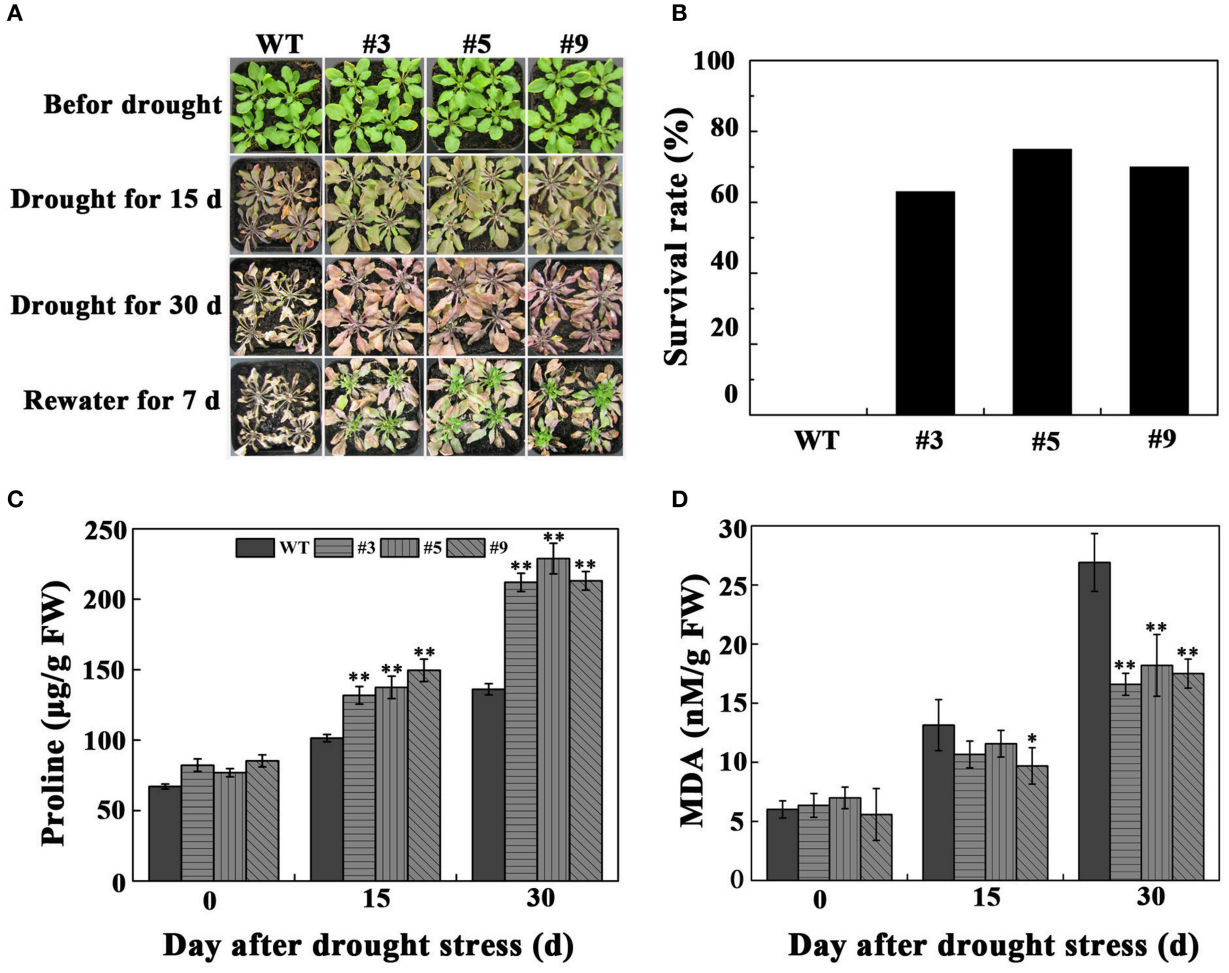
**Photographs of the transgenic lines and WT plants grown in pots under normal and drought conditions. (A)** The effects of water withholding on transgenic lines and WT; **(B)** The survival rates of transgenic and WT plants 7 days after rewatering; **(C)** The content of proline (Pro); **(D)** The content of malondialdehyde (MDA). Each value is the average of three replicates, and *error bars* represent ±SD. ^*^ and ^**^ represent significant differences between transgenic lines and WT at *P* < 0.05 and *P* < 0.01, respectively.

For drought tolerance analysis of *FtbHLH3* transgenic lines in soil, 4-week-old transgenic lines (#3, #5, and #9) and WT were tested. After withholding water for 15 days, all of the leaves of the WT plants were heavily curled while only a few *FtbHLH3* transgenic plants were affected by the water stress and most of them were still green and fully expanded (Figure 6A). After 30 days of water stress, all of the leaves of the WT plants wilted and their leaves became white, whereas the transgenic plants remained turgid and became purple. After 7 days of re-watering, most of transgenic plant leaves became green and grew buds again. The survival rate of the positive lines was significantly higher than WT (Figure 6B).

**Figure 7:**
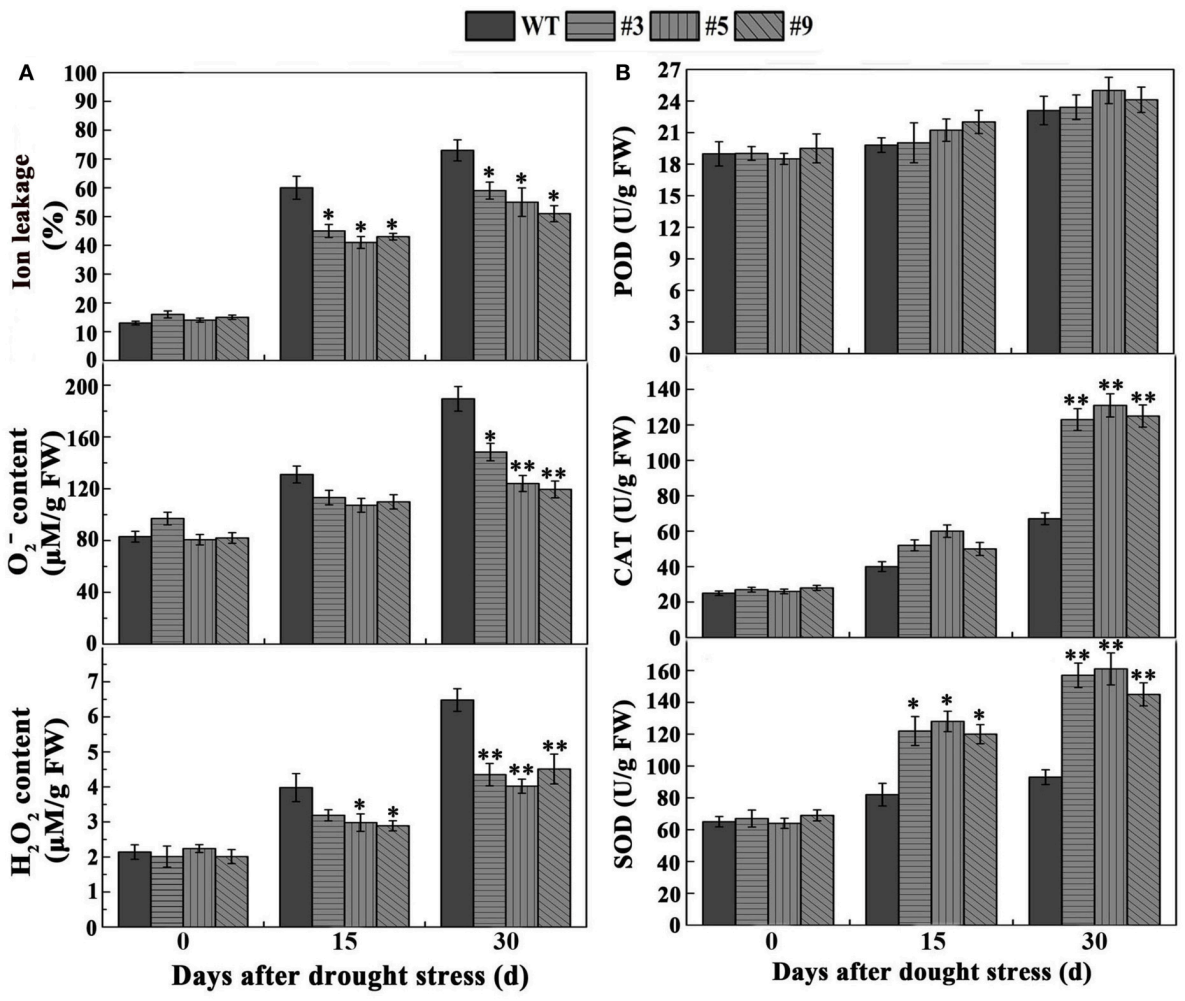
**(A)** The contents of H_2_O_2_, ${\text{O}}_{2}^{-}$ and IL. **(B)** The activities of POD, CAT, SOD in transgenic lines and control under normal and drought conditions. Each value is the average of three replicates, and *error bars* represent ±SD. ^*^ and ^**^ represent significant differences between transgenic lines and WT at *P* < 0.05 and *P* < 0.01, respectively.

To clarify the effect of *FtbHLH3* on physiological indexes of transgenic plants after withholding water, we also measured proline, MDA, and ion leakage (IL). The results showed that proline, MDA, and IL displayed a similar pattern in all of the plants under normal conditions. However, the content of proline was significantly higher than WT plants (Figure 6C). Meanwhile, after drought stress treatment, MDA and IL were significantly lower in the transgenic lines than WT after prolonged stress (Figures 6D,7A). At the beginning of drought stress, the content of ${\text{O}}_{2}^{-}$ and H_2_O_2_ increased in both the experimental and control groups. Over time, the accumulation of ${\text{O}}_{2}^{-}$ and H_2_O_2_ were significantly lower in the transgenic plants than that in WT (Figure 7A). We also detected the activities of three important antioxidant enzymes (POD, CAT, and SOD). Under adequate water conditions, the three antioxidant enzyme activities were not different between the transgenic lines and WT. After drought treatment, the transgenic plants showed higher SOD and CAT activities (Figure 7B). There was no obvious change in POD activity after treatment.

**Figure 8:**
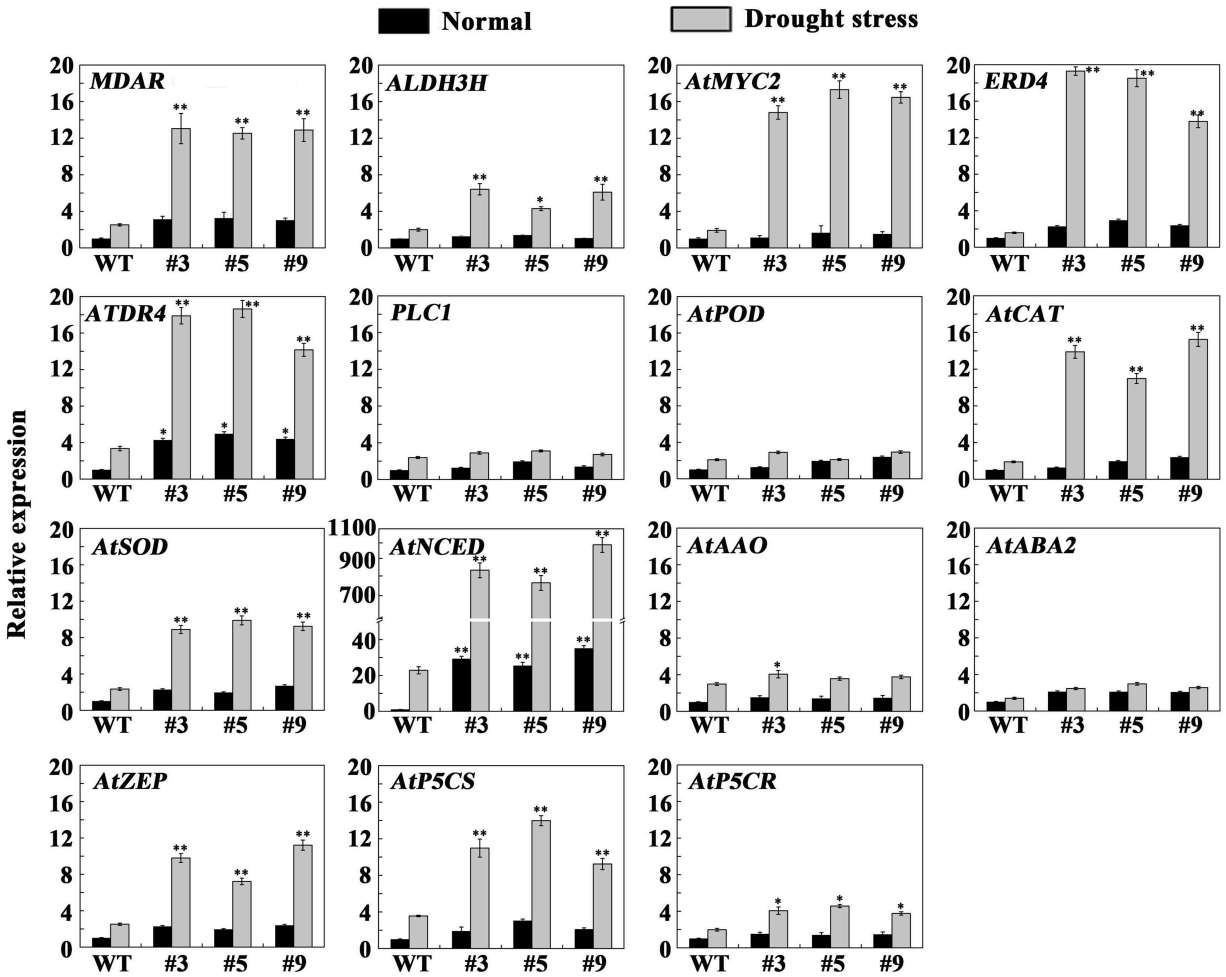
**Analysis of ROS-related, ABA-related, proline-related, and drought stress-responsive genes in transgenic and WT plants under normal and drought stress**. Total RNA was extracted from transgenic lines and WT plants incubated for 30 days under normal or drought stress. The *A. thaliana* actin gene was used as a housekeeping gene. The 2^−ΔΔCT^ method was used to evaluate the relative expression, and the expression levels of genes in the WT plants under normal conditions were defined as “1.” Each value is the average of three replicates, and *error bars* represent ±SD. ^*^ and ^**^ represent significant differences between transgenic lines and WT at *P* < 0.05 and *P* < 0.01, respectively.

To more deeply study the molecular mechanism of *FtbHLH3* in drought stress, the expression levels of several genes were detected in the transgenic and WT plants under normal and drought conditions (Figure 8). Genes selected for this analysis included *AtSOD, AtCAT*, and *AtPOD* involved in ROS detoxification, *AtABA2, AtAAO, AtNCED*, and *AtZEP* involved in ABA biosynthesis, *AtP5CS* and *AtP5CR* involved in proline biosynthesis, and *ERD4, ATDR4, MDAR, ALDH3H1, PLC1*, and *AtMYC2* related to drought stress defense (Wang et al., 2016a). The qPCR results indicated that the expression of *ATDR4* and *AtNCED* was higher in transgenic plants than in the WT plants under the normal conditions, while the other genes had no obvious change. After the drought treatment for 30 days, the tested genes, including *MDAR, ALDH3H, AtMYC2, ERD4, ATDR4, AtCAT, AtSOD, AtNCED, AtZEP*, and *AtP5CS*, showed remarkably increased expression in transgenic *Arabidopsis* compared to WT plants. The data could suggest that FtbHLH3 play an important role in the regulatory mechanism in some ways.

### Overexpression of *FtbHLH3* improves the tolerance of transgenic *Arabidopsis* to oxidative stress

Drought conditions are often accompanied by oxidative stress. MV is a herbicide and is commonly used to simulate oxidative stress, which generates ROS and causes membrane damage and chlorophyll degradation when used to treat the plants (Slade, 1966). As shown in Supplemental Figure S4A, most of the wild-type leaves were bleached while the color of the transgenic lines retained more green under the same concentration. Furthermore, with the increase in MV, the leaves of wild-type plants were injured more seriously, but the transgenic plants showed less injury. The results of the chlorophyll content determination indicated that the positive plants had significantly higher amounts than the wild type (Supplemental Figures S4B,C). In addition, transgenic plants also displayed lower H_2_O_2_ contents than WT (Supplemental Figure S4D). In contrast, higher antisuperoxide anion activity was detected in the transgenic lines (Supplemental Figure S4E).

**Figure 9:**
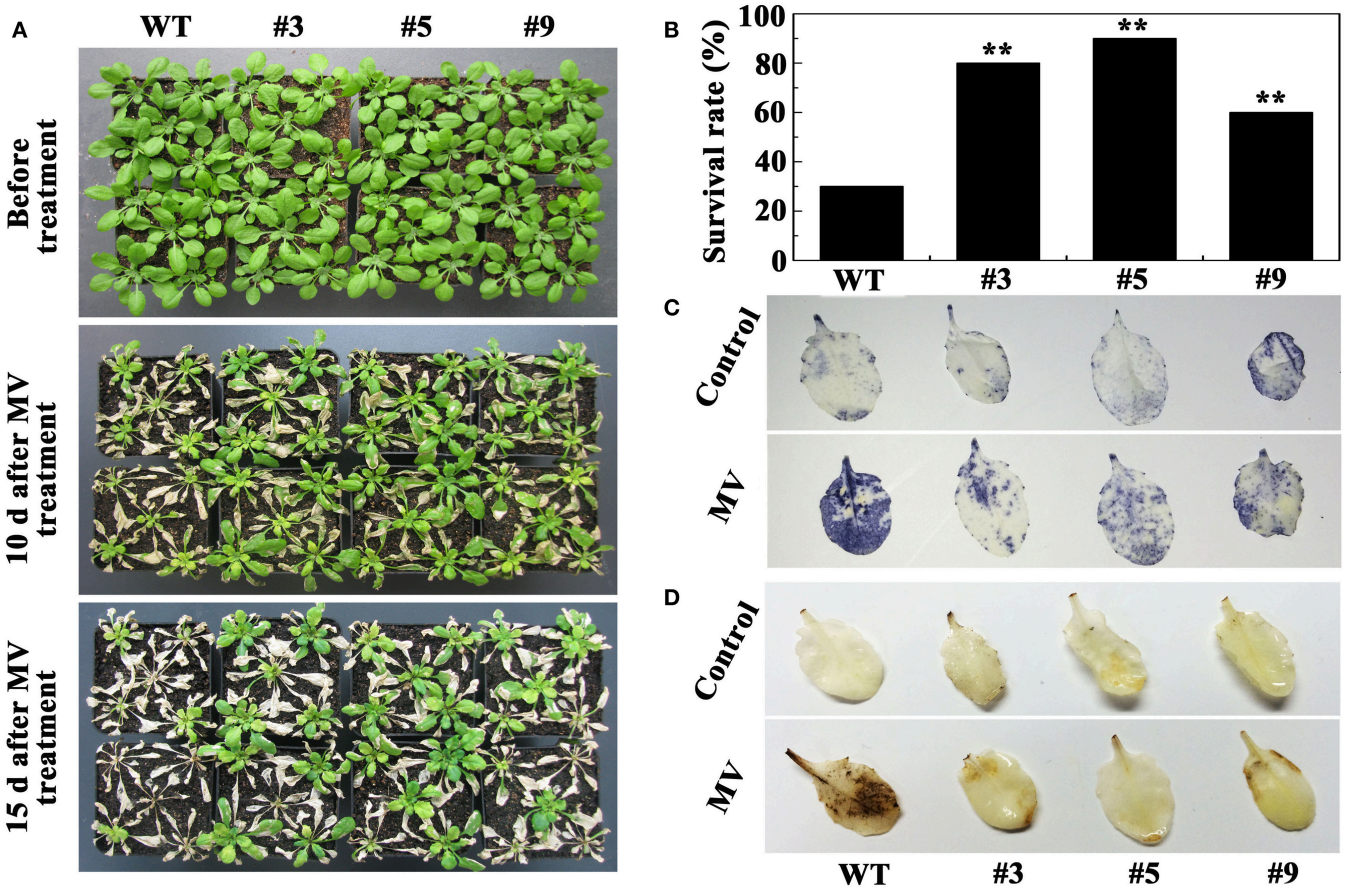
**Oxidative stress analysis of transgenic lines and WT plants. (A)** Phenotypes of 5-week-old *oxFtbHLH3s* and WT plants were treated with 20 μM methyl viologen (MV) for 15 days; **(B)** The survival rates of *oxFtbHLH3s* and WT plants after oxidative stress; **(C,D)** Histochemical staining assays were used to detect ${\text{O}}_{2}^{-}$ and H_2_O_2_ by NBT **(C)** and DAB **(D)** staining, respectively.

**Figure 10:**
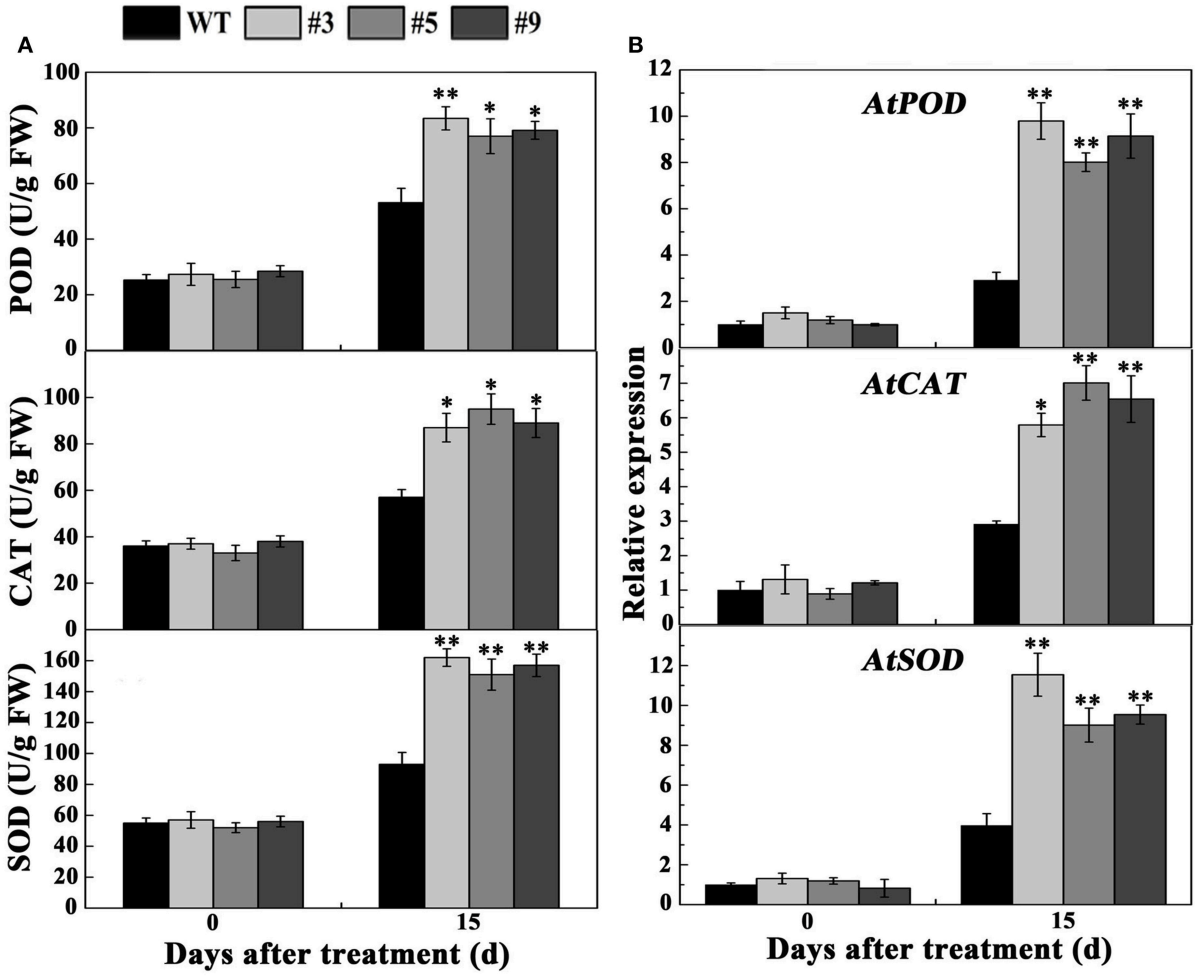
**Determination of activities and expression of oxidative enzymes in transgenic lines and WT plants under normal conditions and 20 μM methyl viologen (MV) treatment**. Five-week-old plants were treated with 20 μM MV for 15 days, and the leaves were used as samples to measure the activities of SOD, CAT, and POD **(A)** and the expression of *AtSOD, AtCAT*, and *AtPOD* **(B)**.

According to the above results, we selected 20 μM MV to treat the plants. Most of the WT plants died, whereas the transgenic lines exhibited better growth phenotypes when 30-day-old *A. thaliana* were treated with MV for 15 days (Figure 9A). The survival rates of the #3, #5, and #9 lines were 80, 90, and 60%, respectively, significantly higher than the 30% of WT (Figure 9B). DAB and NBT staining indicated that the WT plants accumulated more H_2_O_2_ and ${\text{O}}_{2}^{-}$ than the three positive lines (Figures 9C,D). Meanwhile, the activity of three enzymes (POD, CAT, and SOD) increased in the WT and positive lines after oxidative stress; however, the increase in the positive plants was significantly higher (Figure 10A). We also examined the expression of *AtPOD, AtCAT*, and *AtSOD*, and the qRT-PCR analysis results showed that the expression of the three genes in the transgenic plants was significantly higher than in wild type under oxidative stress (Figure 10B).

### Changes in chlorophyll fluorescence under oxidative stress

**Figure 11:**
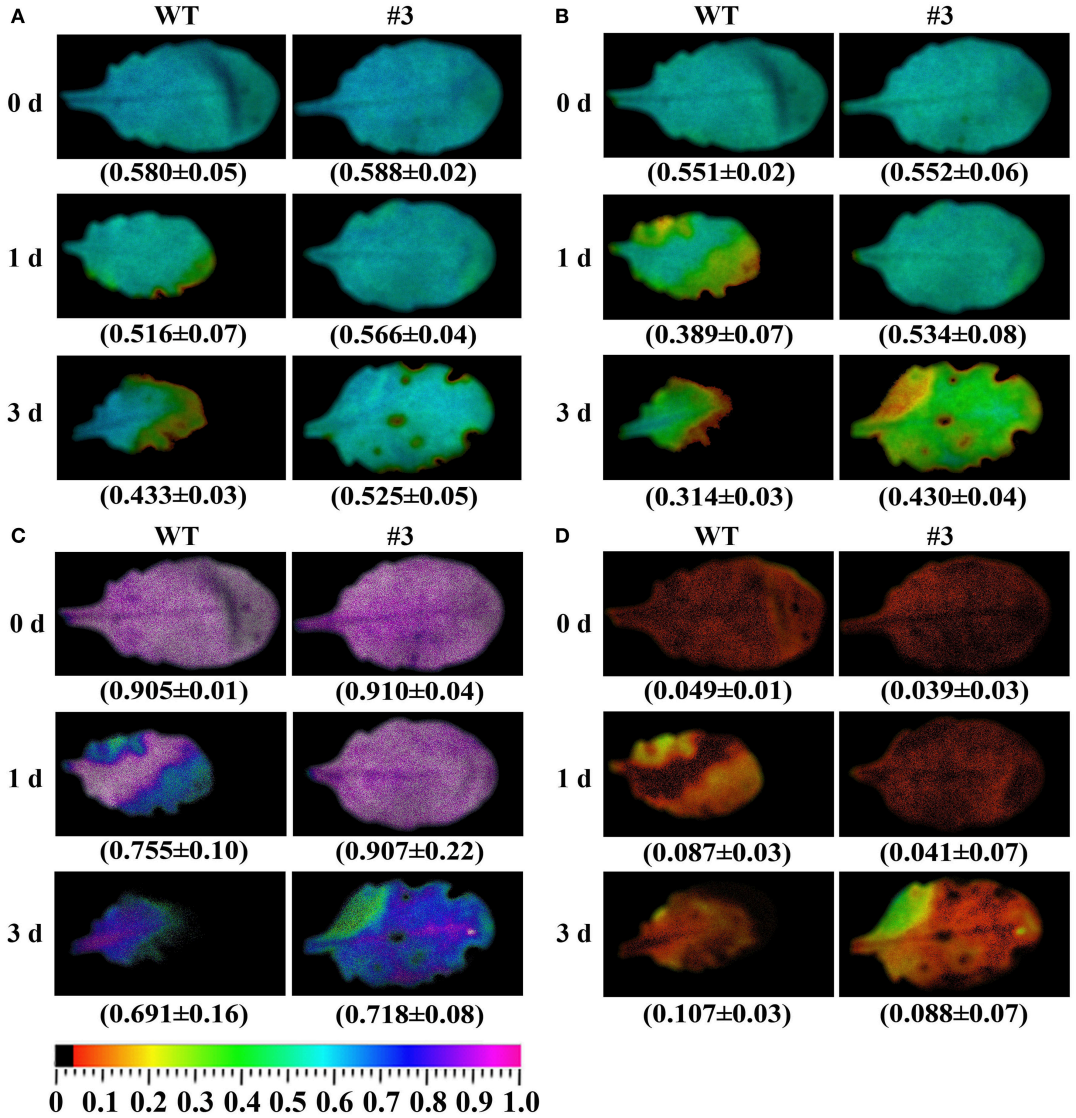
**Effects of methyl viologen (MV) on chlorophyll fluorescence parameters in plants. (A)** *F*v/*F*m, the maximum efficiency of PSII photochemistry; **(B)** ΦPSII, quantum yield of PSII electron transport; **(C)** qP, photochemical quenching; **(D)** NPQ/4, non-photochemical quenching coefficient. Quantitative values (±SD) are shown below the individual fluorescence images. 0–3 days represents MV stress for 0 day (control), 1 day, and 3 day.

Many studies have indicated that photosystem II (PSII) is the most relevant to osmotic stress. To analyze the effects of oxidative stress on PSII photochemistry in transgenic and WT plants, chlorophyll fluorescence images and values were detected using a modulated imaging fluorimeter. There was no significant difference in the color or value of the maximum photochemical efficiency of PSII in the dark-adapted state (*F*v/*F*m), the photochemical quenching (qP), the quantum yield of photosystem II (PSII) photochemistry (ΦPSII), and the non-photochemical quenching coefficient (NPQ) before treatment (Figure 11). Compared with transgenic plants, WT presented a significant decrease in the *F*v/*F*m, ΦPSII, and qP under oxidative stress for 1 and 3 days (Figure 11). Meanwhile, oxidative stress resulted in a significant increase in the level of NPQ in WT compared with transgenic plants.

### Isolation and sequence analysis of the FtbHLH3P

In this study, we cloned a 989 bp promoter fragment upstream of the start codon of *FtbHLH3*. Sequence analysis indicated that multiple *cis*-acting elements involved in abiotic and biotic stress responses were contained in the FtbHLH3P region. For example, there are three copies of the ACGT element, which was reported to be involved in up-regulation of the *erd1* gene in *Arabidopsis thaliana* by drought stress (Simpson et al., 2003). The GATA-box, T-box, and I-box participated in light responsiveness. In addition, many other *cis*-acting elements involved in the regulation of genes under abiotic stresses, such as ABA-response-element (ABRE), cold (DRE2 element), water (MYB), and salicylic acid signaling (W-box), were also observed (Supplemental Figure S5).

**Figure 12:**
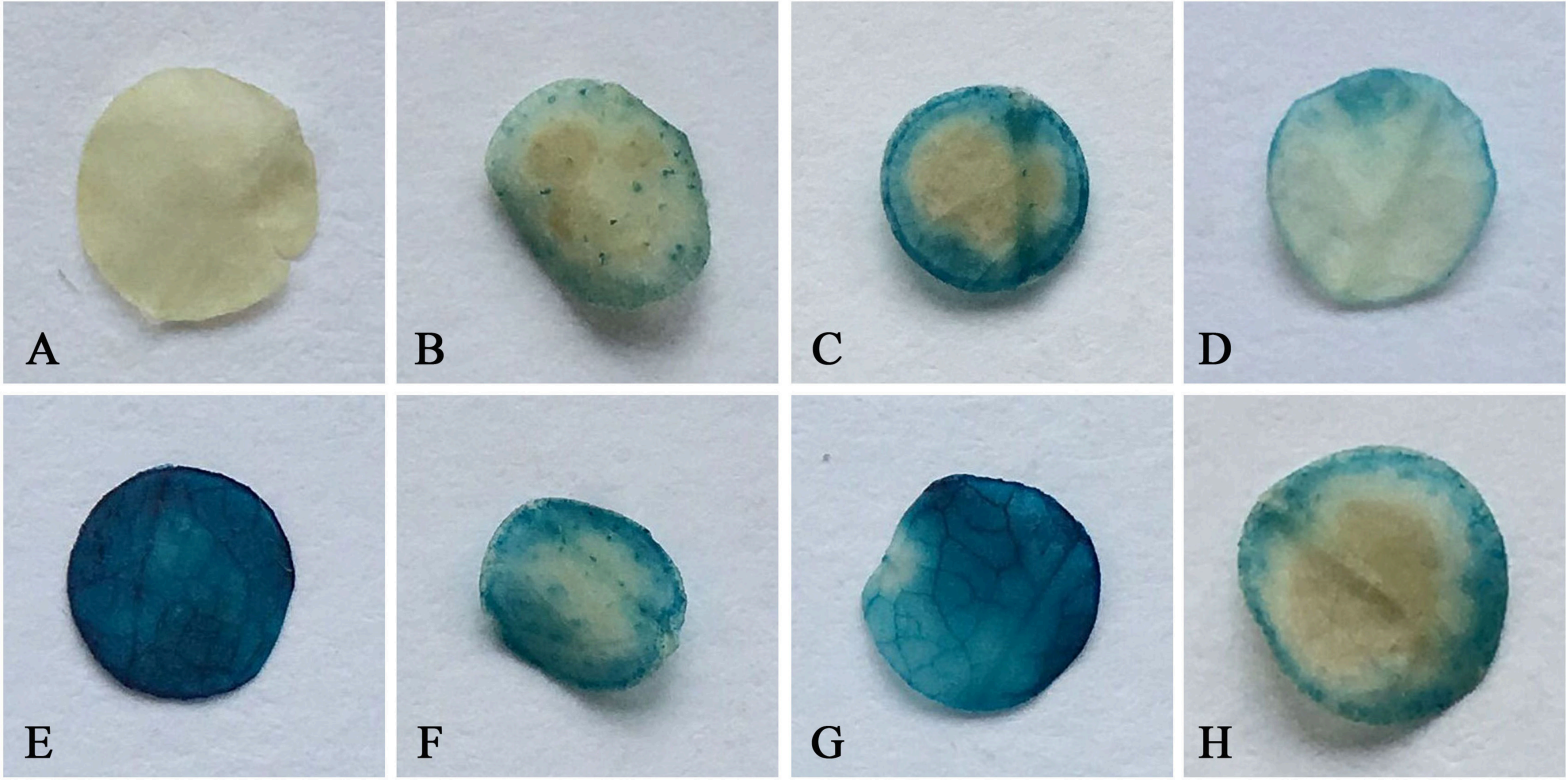
**Histochemical analysis of GUS in transgenic *A. thaliana* plants with the *FtbHLH3* promoter. (A)** Non-transformed plants as negative controls; **(B–D)** positive lines with the *FtbHLH3* promoter under normal conditions; **(E)** Transformed plants with the CaMV 35S promoter as a positive control; **(F–H)** Positive lines with the *FtbHLH3* promoter were subjected to drought treatment for a week.

The activity of FtbHLH3P was tested in *A. thaliana* plants transformed with FtbHLH3P:GUS fusion constructs; non-transformed plants were used as negative controls, and plants transformed with the CaMV 35S promoter were used as positive controls. According to the results of the GUS activity assay, the positive control and transgenic lines with FtbHLH3P stained different degrees of blue (Figure 12). Meanwhile, the GUS histochemical assays were more obvious when transgenic *Arabidopsis* lines were treated with drought treatment.

## Discussion

In plants, TFs, including the bHLH family, function in various pathways to confer stress tolerance. Several plant bHLH factors that have been functionally characterized and identified are involved in responses to abiotic stresses, such as AtbHLH61/93/122 (Zhao et al., 2013; Liu et al., 2014), OrbHLH2 (Zhou et al., 2009), and OsbHLH148 (Seo et al., 2011). In this study, a drought-inducible bHLH TF, FtbHLH3, was identified from tartary buckwheat. Transactivation and sub-localization assays indicated that FtbHLH3 functions as a transcription activator. The multiple sequence alignment results showed that a sequence rich in acidic amino acids is present at the C-terminal region, which was believed to be the transactivation (ACT) domain that interacts with the RNA polymerase II machinery and then initiates transcription (Pattanaik et al., 2008). In addition to this conserved region, several bHLH transcription factors have a MYB interaction site in the N-terminal region (Lai et al., 2016). However, we did not find this site in FtbHLH3, suggesting that the transcription factor may bind independently to its DNA target genes.

In our experiments, *FtbHLH3* responded to multiple abiotic stresses, such as cold, ABA, PEG, and UV-B. However, the response of the *FtbHLH3* gene to each treatment was different, in which PEG stress was the most intense. Generally, transcription factors are induced rapidly in response to abiotic stress during the early stages, reach a maximal induction after several hours, and then decrease in expression level (Dai et al., 2007). For instance, the expression level of *PebHLH35* showed a peak induction at 4 h during stress treatment in *P. euphratica* (Dong et al., 2014). However, *AtbHLH122* reached a maximum at 12 h in wild-type *Arabidopsis* after drought treatment (Liu et al., 2014). Similar to *AtbHLH122*, the transcript levels of *FtbHLH3* accumulated quickly after PEG treatment for 6 h and reached its maximal level at 12 h, then tended to be high with little alteration in tartary buckwheat. This result may be due to the tartary buckwheat itself with a strong fundamental resistance to abiotic stress (Park et al., 2011; Li et al., 2015). Then, the enhanced expression of *FtbHLH3* at a slightly slower pace could still meet the requirements for metabolism stability. However, the expression of *FtbHLH3* increased rapidly to 14.56-fold after drought treatment for 12 h, indicating that *FtbHLH3* should respond to the drought stress in tartary buckwheat. The subsequent transgenic experiments also suggested that *FtbHLH3* could significantly improve the tolerance of transgenic *Arabidopsis* to drought/oxidative stresses.

The different stressful environments usually cause a variety of morphological, physiological, and biochemical changes in plants, which effect their normal growth (Vierling and Kimpel, 1992). Osmotic stress is the earliest challenge of plants under drought conditions, which are manifested primarily in seed germination rate and root length (Manavalan et al., 2009). Overexpression of *AtMYB12* significantly enhanced the tolerance to salt and drought stress in transgenic *Arabidopsis*. Under salt and PEG6000 treatments, the transgenic plants exhibited prominently higher root growth rates than WT plants (Wang et al., 2016a). In this study, The transgenic lines of *FtbHLH3* exhibited longer roots and higher germination rates, similar to *AtbHLH122* (Liu et al., 2014). Additionally, proline is an important osmolyte in plants and is considered one of the compatible osmolytes in combating/ameliorating the detrimental effects of drought stress in many plants (Xiong et al., 2014). The increased tolerance of the transgenic *Arabidopsis* may also be caused by the accumulation of sufficient proline to adjust osmotic pressure during the drought condition compared to the WT plants. Moreover, drought/oxidative stresses are accompanied by the formation of ROS, such as ${\text{O}}_{2}^{-}$ and H_2_O_2_, which damage membranes and macromolecules (Kasukabe et al., 2004). For example, overexpression of *PtrbHLH* significantly decreased the content of endogenous H_2_O_2_ in *Arabidopsis* under oxidative stress (Huang et al., 2013). Here, the results of NBT and DAB staining showed that ROS accumulation was markedly induced in the leaves of wild-type plants (Figures 10C,D), which implied that there is a relationship between increased cell death and ROS accumulation. After drought/oxidative stress treatment, the higher content of ROS in the plants could also be evaluated by the accumulation of MDA and IL (Tu et al., 2016). Due to the lower MDA and IL content, the *FtbHLH3* transgenic plants presented a higher drought resistance. To reduce the excessive ROS accumulation, plants have developed a complex set of antioxidant strategies to eliminate their damaging effects and maintain redox homeostasis (Gill and Tuteja, 2010). In fact, the coordinated action of antioxidant enzymes and antioxidant compounds helps to reduce oxidative damage. Previous studies showed that the increase in antioxidant enzyme activities, including SOD, POD, and CAT, enhanced the resistance of wheat to drought tolerance (Hameed et al., 2012). The SOD, POD, and CAT activities in transgenic *Arabidopsis* were significantly higher than in WT under drought/oxidative stress, although there were no obvious differences between transgenic lines and the WT line under normal conditions. Here, we believe that the protective antioxidant system was activated by *FtbHLH3* in the transgenic lines, which subsequently changed the levels of ROS and avoided severe oxidative damage caused by ROS overproduction.

To uncover the downstream genes of *FtbHLH3*, multiple metabolic pathway genes were analyzed by qPCR in *oxFtbHLH3* plants and WT plants under drought condition. The results demonstrated that several genes, including *MDAR, ALDH3H, AtMYC2, ERD4, ATDR4, AtCAT, AtSOD, AtNCED, AtZEP*, and *AtP5CS*, were significantly up-regulated in transgenic *Arabidopsis* after drought stress. These genes were useful in protecting plants from further damage induced by stresses (Zhu, 2001). Thus, the higher expression of these genes may enhance the stress resistance of *Arabidopsis*. The abiotic stress response signaling transduction pathways can be divided into two major forms: ABA-dependent and ABA-independent pathways (Shinozaki and Yamaguchi-Shinozaki, 2007). In this study, *FtbHLH3* was highly induced by exogenous ABA treatment, and the key genes (*AtNCED* and *AtZEP*) involved in the ABA biosynthesis pathway were also remarkably up-regulated in transgenic lines compared to WT lines under drought stresses. ABA biosynthesis is largely regulated by the rate-limiting enzyme gene *AtNCED* under stress (Huang et al., 2010). These results suggested that *FtbHLH3* may have a protective role *via* ABA-dependent signaling transduction pathways. Indeed, an ABA-response-element (ABRE) was found in the *FtbHLH3* promoter region. Studies have revealed that ABRE *cis*-elements mainly exist in the promoter regions of ABA-responsive genes, which is the major *cis*-element for ABA-responsive gene expression (Maruyama et al., 2012). ABRE-binding protein or TFs usually regulate gene expression through ABA-dependent pathways (Nakashima et al., 2014). Therefore, FtbHLH3 may increase the relative gene expression to improve the transgenic *Arabidopsis* tolerance to drought stress through the ABA-dependent pathway.

Several studies also revealed that the measurement of chlorophyll fluorescence parameters could be used as a rapid and accurate approach to evaluate drought stress tolerance in plants (Ashraf and Harris, 2013). Environmental stresses could lead to a reduction in photosynthetic efficiency, which is typically expressed as a *F*v/*F*m decrease (Sperdouli and Moustakas, 2012). For instance, the overexpression of a drought-induced gene, *OsbHLH148*, enhanced drought tolerance in *Arabidopsis*, and transgenic lines showed a higher *F*v/*F*m compared to the wild type (Seo et al., 2011). Consistent with these previous findings, our results showed that the WT line had a stronger decline of chlorophyll fluorescence than the transgenic lines, suggesting that the transgenic lines accumulated less damage in the oxidative stress conditions. Simultaneously, the higher qP and ΦPSII also reflect the higher quantum yield of PSII in the transgenic *Arabidopsis*, indicating that a large fraction of absorbed irradiance was utilized through the photochemical reaction. However, the NPQ value rises when plants suffer from various environmental stresses. This finding is consistent with the above results obtained from oxidative stress. The higher NPQ in drought-susceptible plants showed the increased need to dissipate excess light energy (Chen et al., 2015). Therefore, these results indicated that transgenic plants develop more effective protective mechanisms for avoiding photosynthetic apparatus damage under drought stress.

**Figure 13:**
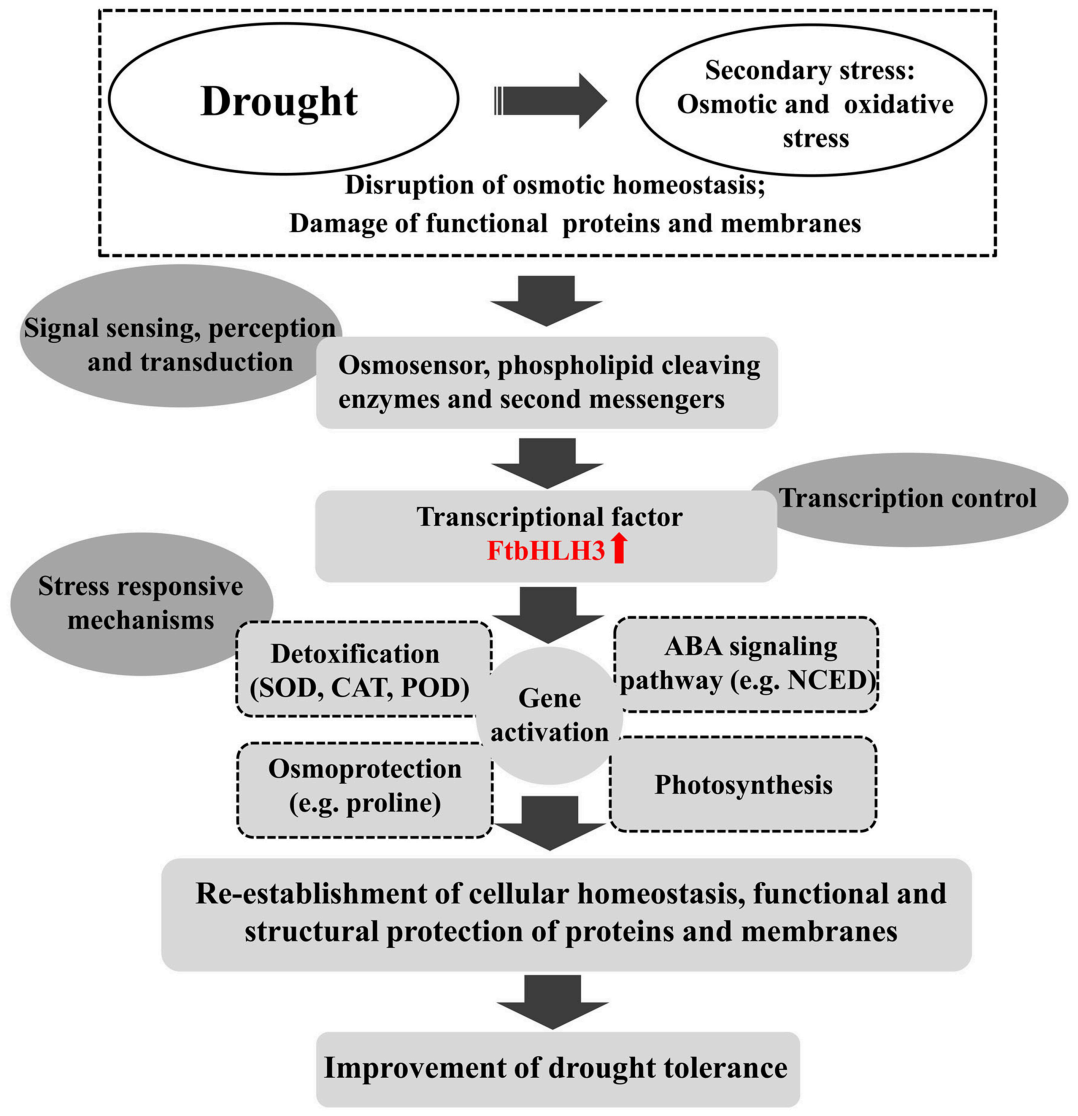
**Hypothetical model of the regulatory network of the FtbHLH3 responses to drought stresses**.

In summary, a drought-related gene, *FtbHLH3*, was isolated from tartary buckwheat and functions as an important TF in enhancing drought stress tolerance in an ABA-dependent pathway. The overexpression of *FtbHLH3* increased the tolerance of transgenic *Arabidopsis* to drought stress by changing the accumulation of several physiological parameters; up-regulating the expression of key genes in the ABA signaling pathway, proline biosynthesis pathway, ROS scavenging system, and drought-responsive pathway; and increasing the photosynthetic efficiency (Figure 13). In conclusion, these findings will provide valuable information toward improving drought-tolerant traits in commercial crop species.

## Author contributions

PY and CL are responsible for most of the experiments and all the data analysis, and PY wrote the draft of the paper. XZ, ML, HZ, and JG carried out part of material collection, RNA extraction. YC and HC participated in the preparation of the manuscript. QW conceived and designed the studies. All authors have read and approved the final manuscript.

### Conflict of interest statement

The authors declare that the research was conducted in the absence of any commercial or financial relationships that could be construed as a potential conflict of interest.

## Supplementary material

Supplemental Figure S1 **Expression profiles of *FtbHLH3* in the root, stem, leaf, and flower were analyzed by qRT-PCR**. *FtH3* was used as a housekeeping gene. The expression of *FtbHLH3* in the leaf was defined as “1” using the 2^−ΔΔCT^ method. *Error bars* represent ±SD. The different lowercase letters represent the significant difference within different organs.

Supplemental Figure S2 **Expression profiles of *FtbHLH3* after 4°C, ABA, NaCl, PEG6000, SA, and UV-B treatments in tartary buckwheat seedlings were analyzed by qRT-PCR**. The expression levels at 0 h (no treated) were set to “1” using the 2^−ΔΔCT^ method. *FtH3* was used as a housekeeping gene. Each value is the average of three replicates, and *error bars* represent ±SD. ^*^ and ^**^ represent significant differences between the control and stressed tartary buckwheat at *P* < 0.05 and *P* < 0.01, respectively.

Supplemental Figure S3 **Molecular analyses of the *FtbHLH3*-overexpressing Arabidopsis. (A)** PCR analysis of transgenic plants. *Lane M* DNA marker III; *Lane W* water as a negative control; *Lane P* plasmid pCAMBIA1301- *FtbHLH3* as a positive control; *Lane CK* wild type; *Lanes #1-#10* transgenic plants. **(B)** Expression analysis of *FtbHLH3* in transgenic *Arabidopsis* lines and CK plants. The *Arabidopsis* action gene was used as an internal control. Each value is the average of three replicates, and *error bars* represent ±SD. ^*^*P* < 0.05 and ^**^*P* < 0.01 indicate significant differences between each line, respectively.

Supplemental Figure S4 **Oxidative stress analysis of WT and transgenic plants *in vitro*. (A)** Photograph showing 4-week-old plant leaves subjected to different concentrations of methyl viologen (MV) (10, 20, and 30 μM) on MS medium for 32 h in the light; **(B,C)** Chlorophyll extraction solutions **(B)** and chlorophyll contents **(C)** in the leaves of WT and transgenic plants; **(D,E)** H_2_O_2_ contents **(D)** and antisuperoxide anion activity **(E)** in the leaves of WT and transgenic plants. Each value is the average of three replicates, and *error bars* represent ±SD. ^*^ and ^**^ represent significant differences between transgenic lines and WT at *P* < 0.05 and *P* < 0.01, respectively.

Supplemental Figure S5 **Promoter sequence of the *FtbHLH3* gene**. Potentially functional elements were marked with an underline, box, and gray color, and their names are indicated above.

Supplementary Table S1 **Primers used in this study**.
