# Supplementary figures and images for: Overexpression of a Tartary Buckwheat Gene, FtbHLH3, Enhances Drought/Oxidative Stress Tolerance in Transgenic Arabidopsis
Source: Front Plant Sci. 2017 Apr 25;8:625. doi: 10.3389/fpls.2017.00625 (PMC5403918; doi:10.3389/fpls.2017.00625)

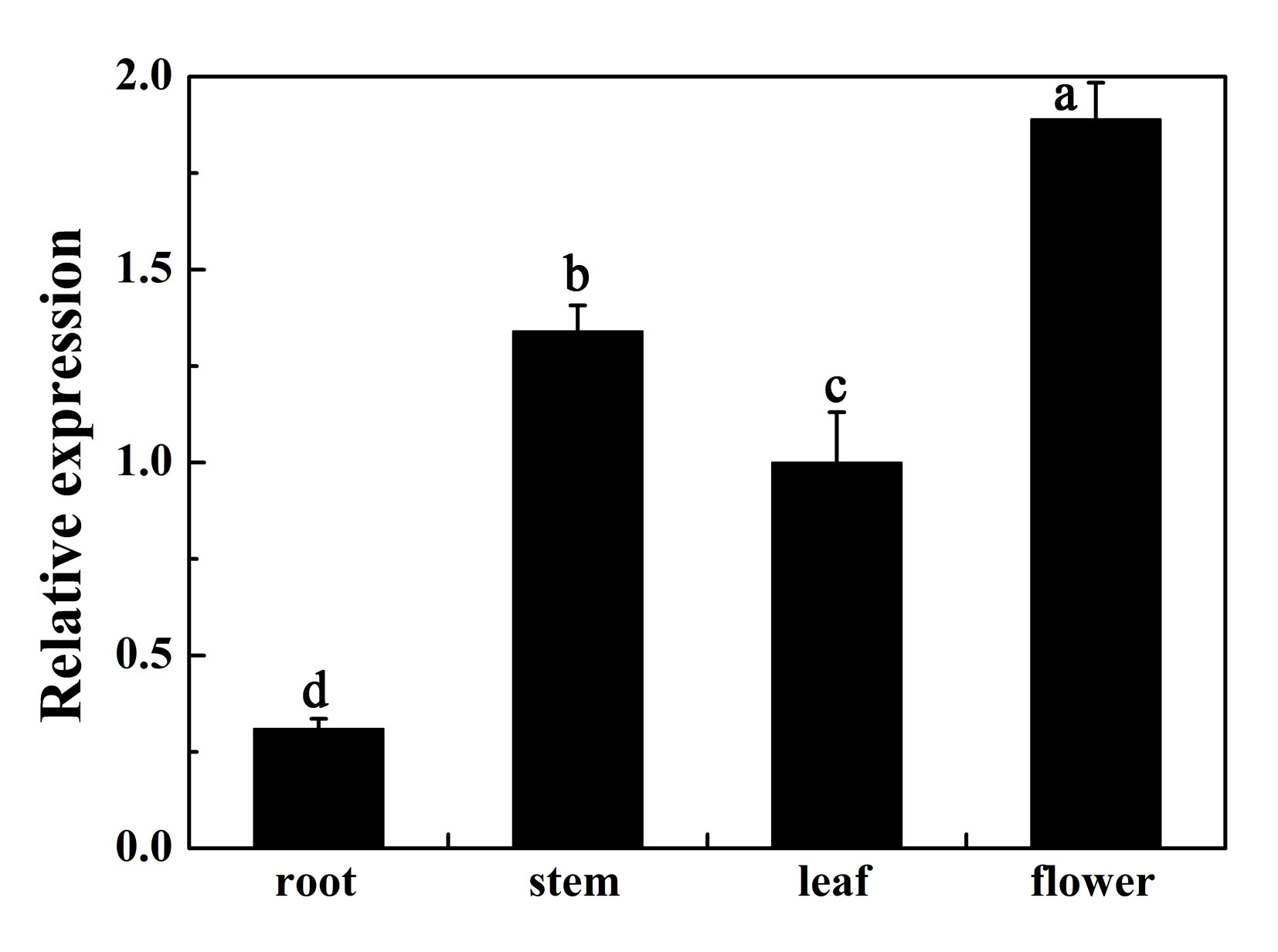

Supplement: Supplemental Figure S1 — Expression profiles of FtbHLH3 in the root, stem, leaf, and flower were analyzed by qRT-PCR. FtH3 was used as a housekeeping gene. The expression of FtbHLH3 in the leaf was defined as “1” using the 2−ΔΔCT method. Error bars represent ±SD. The different lowercase letters represent the significant difference within different organs. [file Image1.JPEG]

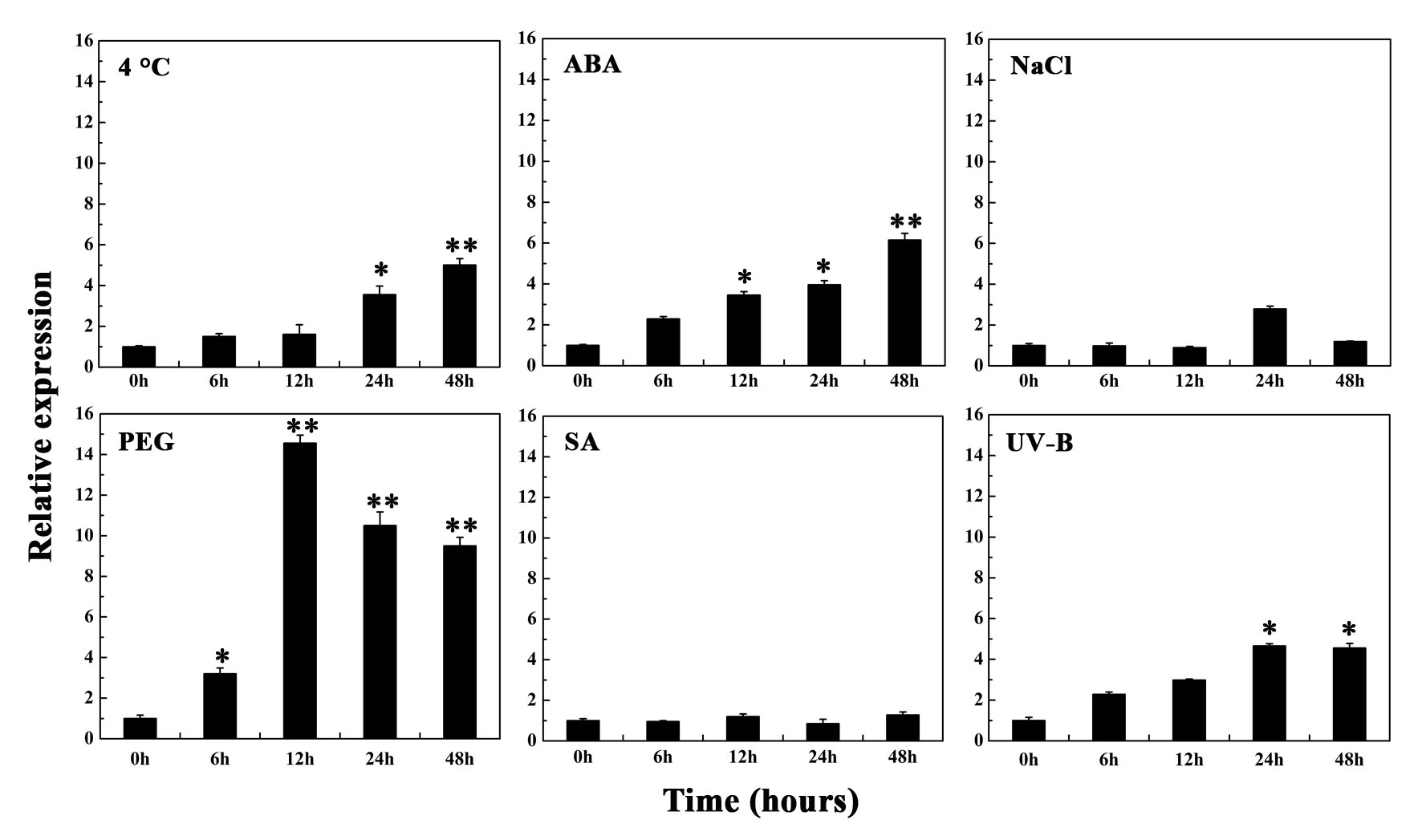

Supplement: Supplemental Figure S2 — Expression profiles of FtbHLH3 after 4°C, ABA, NaCl, PEG6000, SA, and UV-B treatments in tartary buckwheat seedlings were analyzed by qRT-PCR. The expression levels at 0 h (no treated) were set to “1” using the 2−ΔΔCT method. FtH3 was used as a housekeeping gene. Each value is the average of three replicates, and error bars represent ±SD. * and ** represent significant differences between the control and stressed tartary buckwheat at P < 0.05 and P < 0.01, respectively. [file Image2.JPEG]

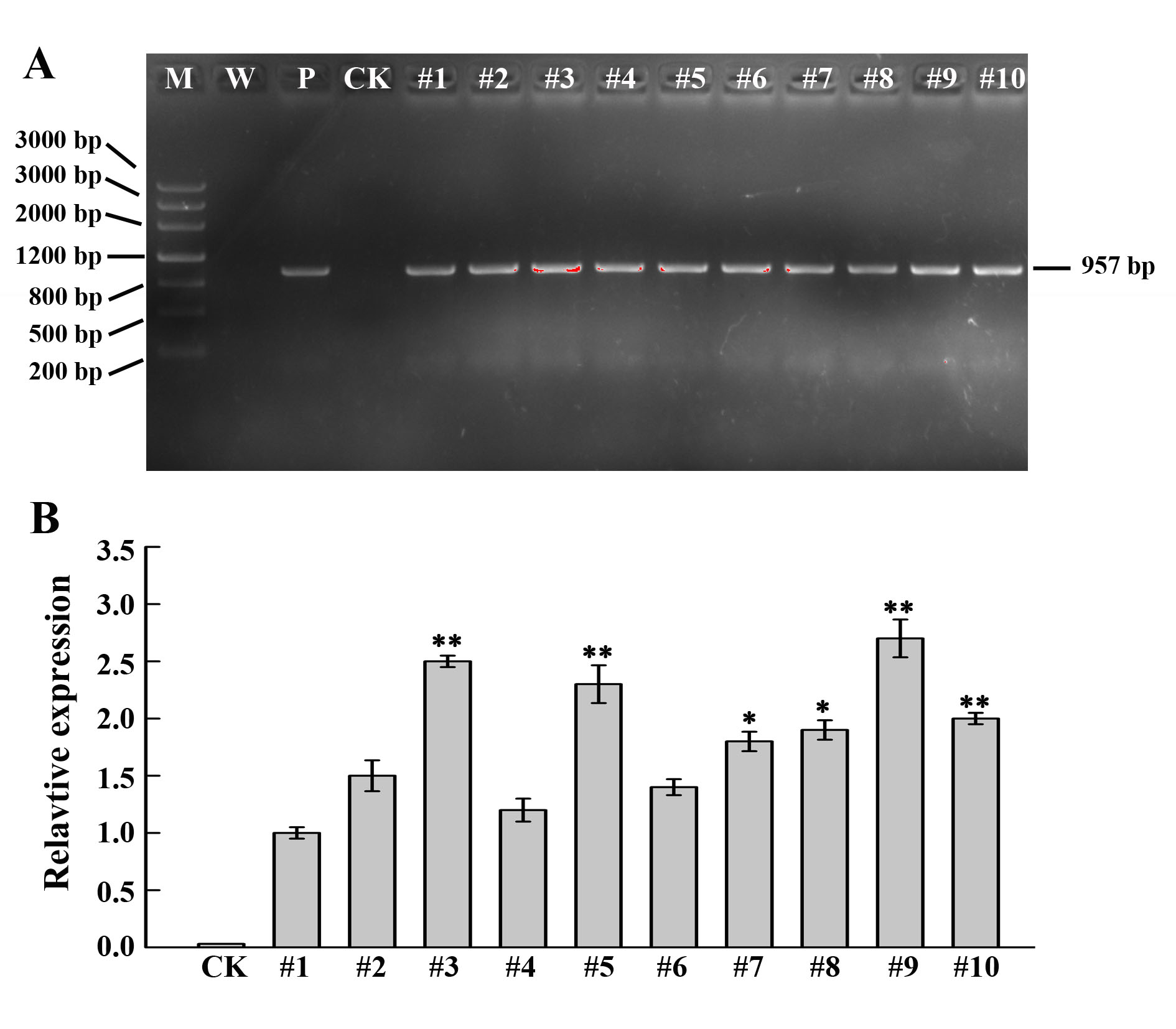

Supplement: Supplemental Figure S3 — Molecular analyses of the FtbHLH3-overexpressing Arabidopsis. (A) PCR analysis of transgenic plants. Lane M DNA marker III; Lane W water as a negative control; Lane P plasmid pCAMBIA1301- FtbHLH3 as a positive control; Lane CK wild type; Lanes #1-#10 transgenic plants. (B) Expression analysis of FtbHLH3 in transgenic Arabidopsis lines and CK plants. The Arabidopsis action gene was used as an internal control. Each value is the average of three replicates, and error bars represent ±SD. *P < 0.05 and **P < 0.01 indicate significant differences between each line, respectively. [file Image3.JPEG]

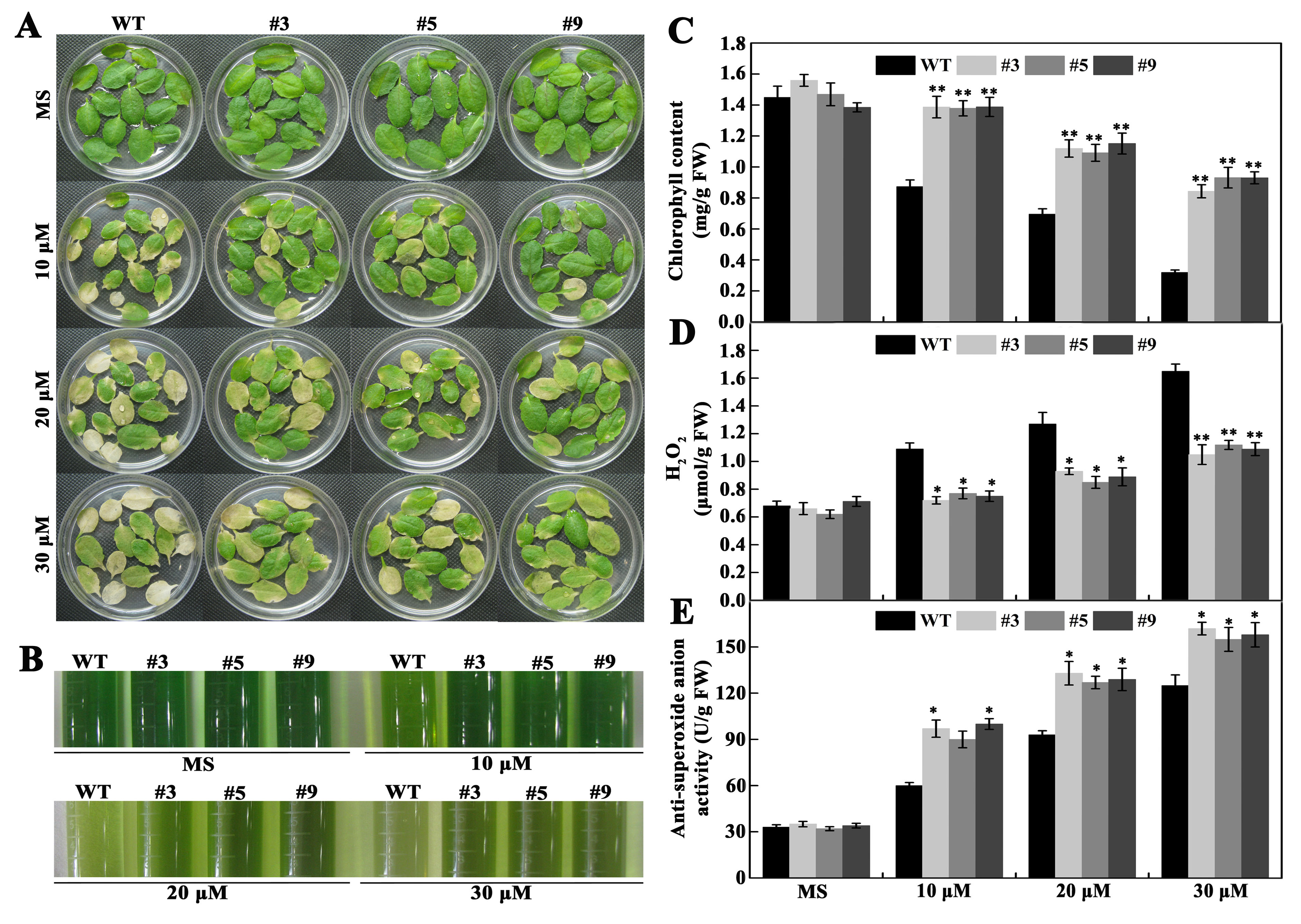

Supplement: Supplemental Figure S4 — Oxidative stress analysis of WT and transgenic plants in vitro. (A) Photograph showing 4-week-old plant leaves subjected to different concentrations of methyl viologen (MV) (10, 20, and 30 μM) on MS medium for 32 h in the light; (B,C) Chlorophyll extraction solutions (B) and chlorophyll contents (C) in the leaves of WT and transgenic plants; (D,E) H2O2 contents (D) and antisuperoxide anion activity (E) in the leaves of WT and transgenic plants. Each value is the average of three replicates, and error bars represent ±SD. * and ** represent significant differences between transgenic lines and WT at P < 0.05 and P < 0.01, respectively. [file Image4.JPEG]

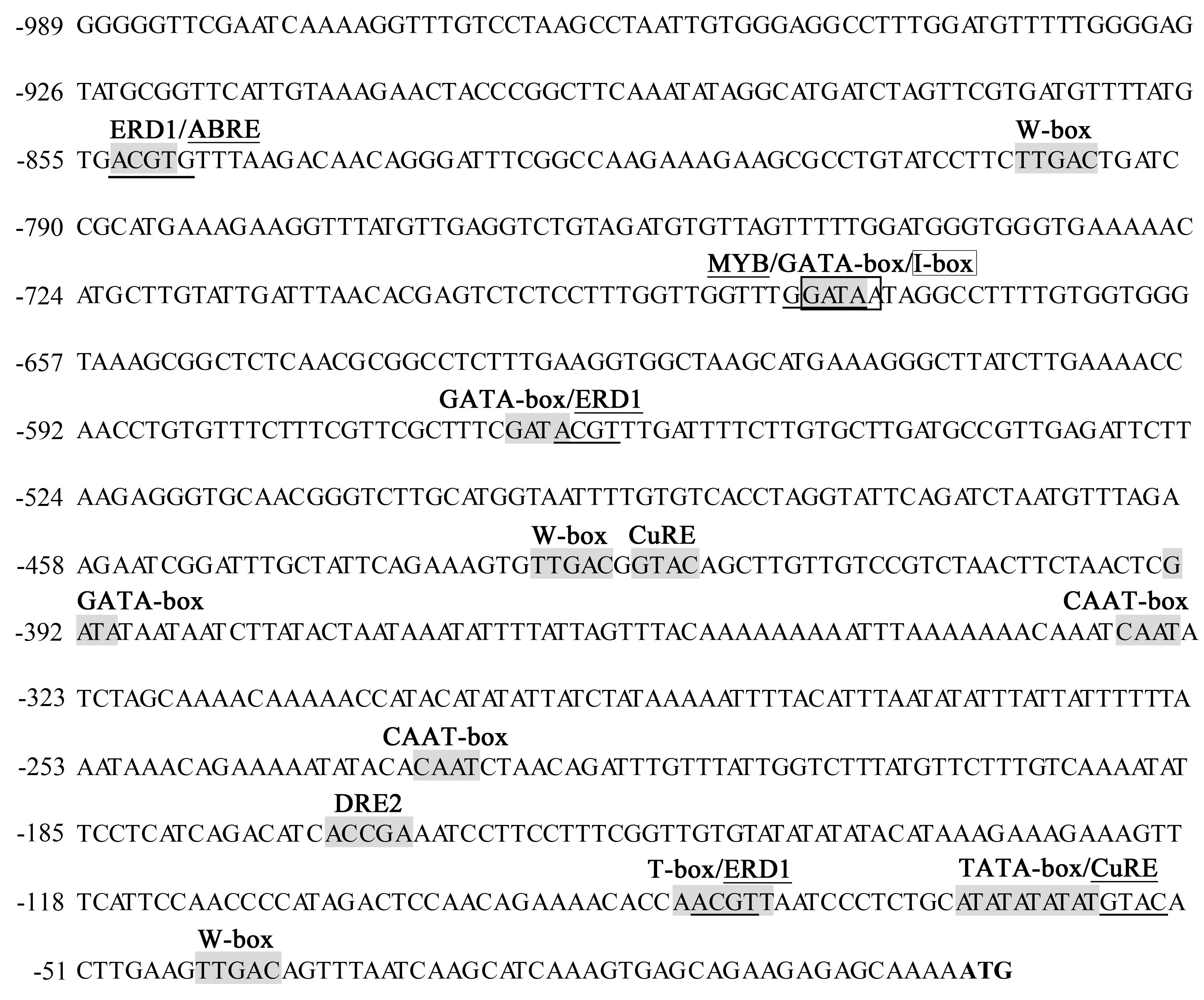

Supplement: Supplemental Figure S5 — Promoter sequence of the FtbHLH3 gene. Potentially functional elements were marked with an underline, box, and gray color, and their names are indicated above. [file Image5.JPEG]
