# Supplementary material for: Overexpression of a Tartary Buckwheat Gene, FtbHLH3, Enhances Drought/Oxidative Stress Tolerance in Transgenic Arabidopsis
Source: Front Plant Sci. 2017 Apr 25;8:625. doi: 10.3389/fpls.2017.00625 (PMC5403918; doi:10.3389/fpls.2017.00625)
Supplement: Supplementary Table S1 — Primers used in this study. [file Table1.DOC]

Supplementary Table S1 Primers used in this study

| Primer name | Primer sequence (5′-3′) |
| --- | --- |
| Primers for clone of *FtbHLH3* gene | |
| *FtbHLH3*-F | ATGGAGGTAAATGAAGATGGGTTT |
| *FtbHLH3*-R | CATATACTTTCCTCCATAACCTGC |
| Primers for subcellular localization of FtbHLH3 | |
| *FtbHLH3*-GFP-F | CGGGATCCATGGAGGTAAATGAAGATGGGTTTT |
| *FtbHLH3*-GFP-R | GCGTCGACCATATACTTTCCTCCATAACCTGCA |
| Primers for Transcriptional assay of FtbHLH3 | |
| *FtbHLH3*-pBridge-F | CGCGGATCCATGGAGGTAAATGAAGATGGGTTTTTG |
| *FtbHLH3*-pBridge-R | AACTGCAGCATATACTTTCCTCCATAACCTGCATT |
| Primers for constructing expression vector | |
| *FtbHLH3*-1301-F | GGGGTACCATGGAGGTAAATGAAGATGGGTTTTTG |
| *FtbHLH3*-1301-R | CGGGATCCCATATACTTTCCTCCATAACCTGCATT |
| Primers for clone of *FtbHLH3* promoter | |
| SP1 | TGTTAAAACTCGGGATTTCCATGGT |
| SP2 | GCGTTTCGAGCTTCTTGAGATTATG |
| SP3 | GGTCTAATTGGGGAAGAAAAGCACT |
| Primers for constructing expression vector | |
| FtbHLH3P-pBI101-F | CCCAAGCTTTGACGTGTTTAAGACAACAGGGAT |
| FtbHLH3P-pBI101-R | CGGGATCCCATTTTTGCTCTCTTCTGCTCACT |
| Primers for real-time quantitative PCR | |
| *FtbHLH3*-F | ATGGAGGTAAATGAAGATGGGTTTT |
| *FtbHLH3*-R | CTACATATACTTTCCTCCATAACCTGC |
| *FtH3*-F | GAAATTCGCAAGTACCAGAAGAG |
| *FtH3*-R | GAAATTCGCAAGTACCAGAAGAG |
| *ERD4-F* | TGCCTGTGAACTGCTTTGGA |
| *ERD4-R* | GCCATTGTGAGCGGACTTTG |
| *ATDR4*-F | CGTCCTGGTCAAACGTACCA |
| *ATDR4*-R | TCGGAGCCTCAAACTCGATG |
| *MDAR*-F | GTACACCGGTTTTTCACCGC |
| *MDAR*-R | CCGACACCAGCGACAACTAT |
| *ALDH3H1*-F | TCCGCTGGAGGCATAGTAGT |
| *ALDH3H1*-R | GTATGGCGGATACCTGACGG |
| *PLC1*-F | AACCGCAAAGGAGGGTTGAA |
| *PLC1*-R | TCCGCTGCGTGAACTTTACT |
| *AtMYC2*-F | TCGTTACTCGGTGACGCAAT |
| *AtMYC2*-R | CCTCCACTAGCACTCGCTTT |
| *AtABA2*-F | ATTGATCACTGGAGGAGCCACAG |
| *AtABA2*-R | ATTACGAATATCAGGGCACGGTG |
| *AtAAO*-F | CAACAGCCATGTTGATACCG |
| *AtAAO*-R | TCTTTGACCTGCACATCGAG |
| *AtNCED*-F | CGCCGGTTTAGTTTATTTCAATGGT |
| *AtNCED*-R | AATCGTACCGACCCGAAGTTTCTAA |
| *AtZEP*-F | CGGAGCTTTCTTCTTGATGG |
| *AtZEP*-R | TCGATTTCGGAGTTTTCCTG |
| *AtP5CR*-F | AGTTTAGCTTCACAGACCGTTC |
| *AtP5CR*-R | GCTCTGTGAGAGCTCGCGGCTTC |
| *AtP5CS*-F | ATGATCTTATTTATGTTCTGC |
| *AtP5CS*-R | CACTATCTTCCGTCACTAT |
| *AtSOD*-F | ATGAGAAGTTCTATGAAGAG |
| *AtSOD*-R | GTCTTTATGTAATCTGGT |
| *AtCAT*-F | GCAACTACCCCGAGTGGAAA |
| *AtCAT*-R | TGTTCAGAACCAAGCGACCA |
| *AtPOD*-F | TCCGGGAGCCACACCATTGG |
| *AtPOD*-R | TGGTCGGAATTCAACAG |
| *Atactin*-F | GCACCCTGTTCTTCTTACCGA |
| *Atactin*-R | AGTAAGGTCACGTCCAGCAAGG |

Enzyme cleavage site were underlined.
